# Balancing boundaries: Observed parental autonomy support and psychological control in the context of parent‐adolescent interactions and adolescent depression

**DOI:** 10.1111/jora.70003

**Published:** 2025-01-26

**Authors:** Wilma G. M. Wentholt, E. H. Alet Meurs, Loes H. C. Janssen, Lisanne A. E. M. van Houtum, Mirjam C. M. Wever, Marieke S. Tollenaar, Lenneke R. A. Alink, Bernet M. Elzinga

**Affiliations:** ^1^ Department of Clinical Psychology, Institute of Psychology Leiden University Leiden The Netherlands; ^2^ Leiden Institute for Brain and Cognition Leiden University Leiden The Netherlands; ^3^ Department of Child, Youth and Psychotrauma GGZ Rivierduinen Leiden The Netherlands; ^4^ Department of Child and Adolescent Psychiatry/Psychology, Erasmus MC University Medical Centre Rotterdam – Sophia Rotterdam The Netherlands; ^5^ Institute of Education and Child Studies Leiden University Leiden The Netherlands

**Keywords:** adolescent depression, observed and perceived parenting, parental autonomy support and psychological control

## Abstract

Autonomy support (AS) and psychological control (PC) are important parenting behaviors in adolescence, with low AS and high PC relating to adolescent depression. Studies on observed levels of AS and PC in a clinical sample are lacking. The current study aimed to (1) develop a reliable coding system for parental AS and PC in parent‐adolescent interactions and gain insights into its ecological validity in a healthy control (HC) sample, and (2) disentangle observed and adolescent‐perceived parenting behaviors in relation to adolescent depression. HC adolescents (*N* = 80, *M*_age_ = 15.90, 63.7% girls, 91.3% White) and their parents (*N* = 148, *M*_age_ = 49.00, 53.4% female, 97.3% White) and adolescents with depression (current MDD/dysthymia; *N* = 35, *M*_age_ = 15.60, 77.1% girls, 65.7% White) and their parents (*N* = 62, *M*_age_ = 50.13, 56.5% female, 79.0% White) participated in three videotaped dyadic interaction tasks (problem solving, event planning, and reminiscence). Adolescents reported on their parents' behavior and their own positive and negative affect after each task, while observed AS and PC were coded from the videos. Multilevel analyses showed that observed AS and PC, coded with our reliable system, related to adolescent‐perceived parenting (in daily life), confirming ecological validity. Adolescents with depression (vs. HC) had more negative perceptions of parenting, whereas observed AS and PC did not differ, indicating a negativity bias of adolescents with depression. Lastly, observed PC related to a lower affective state in adolescents with depression, but not HC. Parents could be psycho‐educated on the impact of this behavior in a clinical setting.

## INTRODUCTION

Humans develop from a state of full dependency on available caregivers in infancy to autonomous individuals over the course of decades (McCurdy et al., 2020). Following the self‐determination theory, autonomy can be defined as the need for independent and purposeful functioning out of intrinsic motivation. It is considered to be one of the basic psychological needs for optimal human development (Ryan & Deci, 2000) and a central developmental task in adolescence (McCurdy et al., 2020; Smetana, 2010). The development of autonomy does not occur in isolation; parental autonomy support is of profound importance across the full span of child development (Vasquez et al., 2016), with different nuances depending on the developmental phase (McCurdy et al., 2020). Specifically during adolescence, parental autonomy support should encourage the adolescent's self‐endorsed decision‐making in which the adolescent can align their own actions and value‐systems (McCurdy et al., 2020). Autonomy supportive behavior is characterized by parents showing structure (i.e., (non)verbal encouragements and patience) and support (i.e., actively accepting of and further exploration of adolescent's input) towards their child's communication, emotions, cognitions, and decision‐making. Importantly, autonomy supportive behavior does not mean that parents do not set any boundaries, but that they do so by clearly and respectfully explaining why they make certain decisions (Deci & Ryan, 2000; McCurdy et al., 2020; Soenens & Vansteenkiste, 2010). Empirical studies show that parental autonomy support positively relates to adolescents' autonomous functioning and broader mental health (meta‐analysis by Vasquez et al., 2016).

In contrast to autonomy support, parental psychological control can be detrimental to development of autonomy. The focus of psychological control lies with parents' attempts to force their child into a parent‐directed perspective. The concepts of autonomy support and psychological control are clearly related, but not mutually exclusive, and therefore reflect separate constructs rather than two ends of the same continuum (Barber et al., 2005; Hauser Kunz & Grych, 2013). Psychologically controlling behavior is characterized by the use of controlling and manipulative tactics (e.g., constraining expressions, guilt induction, invalidation of feelings) that are intrusive towards the child's feelings and thoughts (Barber et al., 2005; Deci & Ryan, 2000; Soenens & Vansteenkiste, 2010). Empirical studies show that parental psychological control negatively relates to adolescents' autonomous functioning and broader mental health (Chyung et al., 2022; Costa et al., 2016; Hare et al., 2015; Yan et al., 2020).

While research highlights the importance of parental autonomy support and psychological control, virtually all studies rely on child/adolescent reports (McCurdy et al., 2020; Vasquez et al., 2016; Yan et al., 2020) and little is known about the relation of observed parental autonomy support and psychological control with adolescent well‐being. There is a need for a coding system for observed autonomy support and psychological control that can be used in parent‐adolescent interaction research, to disentangle observed behaviors from adolescents' perceptions. Three studies included observed parental autonomy support (Wuyts et al., 2018), psychological control (Barber, 1996), and autonomy *granting* and psychological control (Hauser Kunz & Grych, 2013). However, none of these studies included measures of observed autonomy support and psychological control (see Measures). In the current study, we aim to develop a reliable coding system for observed parental autonomy support and psychological control in parent‐adolescent interactions, and gain insights into its ecological validity in families with an adolescent without psychopathology (i.e., healthy control; HC). Ultimately, we aim to provide a coding system that can be used in parent‐adolescent interaction research.

To gain insights into the ecological validity of assessing observed autonomy support and psychological control in the lab setting, we will test whether observed autonomy support and psychological control relate to adolescents' experiences of parenting behaviors (1) in the same interaction and (2) in daily life context. To the best of our knowledge, there are no studies that included (any type of) observed parenting behaviors as well as adolescents' experiences during the same parent–child interaction. However, the study by Wuyts et al. (2018) shows there is a small, albeit significant, correlation between observed and overall adolescent‐perceived autonomy support. A pioneering study using Ecological Momentary Assessment (EMA; also known as Experience Sampling Method) showed that observed parental affect in the lab while interacting with their child, relates to adolescents' perceptions of parental affect in daily life (Griffith et al., 2018). Based on these studies, we expect observed autonomy support and psychological control to relate to some extent to adolescent‐perceived parenting behaviors in the lab and in daily life. In the current study (that is part of a larger research project, see Participants) we used adolescent reports about their parents' listening and understanding to indicate parental autonomy support, and adolescent reports about their parents' dominance and criticism to indicate parental psychological control. With regards to autonomy support, the receptive aspects are covered by asking about listening, and stimulation of adolescents' input is at least partly covered by asking about understanding (though explaining motivations and asking in‐depth follow‐up questions are not explicitly covered). With regards to psychological control, the constraining, invalidating, and criticizing aspects are largely covered by asking about criticism and dominance. However, guilt induction is not covered with these questions, this concept is difficult to assess with a brief questionnaire about parenting in between interaction tasks.

We will further examine observed autonomy support and psychological control in relation to adolescents' affect during the interaction with their parent. Previous studies using EMA show that adolescent‐perceived autonomy support and psychological control (van der Kaap‐Deeder et al., 2023) and other parenting behaviors (e.g., Bülow et al., 2022; Griffith et al., 2018; Griffith & Hankin, 2021; Janssen et al., 2021; Richmond et al., 2013) relate to adolescent affective state in daily life. However, so far, there are no studies linking observed parenting behaviors to adolescent affective state in the lab. The current study will thus add to existing research by linking observed autonomy support and psychological control in the lab to the adolescents' experiences of parenting behavior (in daily life) and to adolescent affect.

The coding system for observed autonomy support and psychological control is used in three dyadic parent‐adolescent interaction tasks (problem solving, event planning, and reminiscence task; see Procedure) to simulate common communication topics between parents and adolescents in daily life. Previous studies showed the importance of context, with more negative observed parenting behaviors in a more challenging (i.e., demanding, stressful) context (Branger et al., 2019; Grolnick et al., 2002; McCurdy et al., 2020). In the current study we will test whether observed and adolescent‐perceived parenting behaviors are more negative in a more challenging context (i.e., problem solving vs. event planning and reminiscence task). The impact of the context on adolescent‐perceived, in addition to observed, parenting behaviors is included to understand whether the context similarly matters for these different perspectives. We will thereby gain insights into the behavior that is elicited (and perceived) by the specific tasks, which can help researchers and clinicians choose the task most relevant to their aim.

### Parental autonomy support and psychological control in the context of adolescent depression

Parental autonomy support and psychological control relate to adolescent mental health in general (Chyung et al., 2022; Vasquez et al., 2016; Yan et al., 2020), but may be particularly relevant in the context of adolescent depression. The prevalence of (clinical) depression increases during adolescence (Lewinsohn et al., 1998; Ormel et al., 2015; Solmi et al., 2022). Adolescent depression is characterized by irritability and negative self‐perceptions (Crowe et al., 2006; Nardi et al., 2013; Orchard et al., 2017; Parker & Roy, 2001), and accompanied by cognitive and somatic symptoms (APA, 2013). Symptoms can substantially impair adolescents in the social, academic, and/or family domain (Clayborne et al., 2019). An adolescent with depression may struggle with negative thoughts about themselves and the world around them, and experience difficulties to get out of bed, go to school, and spend meaningful time with their family.

Empirical studies show that a lack of (perceived) parental autonomy support and the presence of (perceived) parental psychological control can precede, co‐occur, and follow adolescent depression (Barber et al., 2005; Chyung et al., 2022; Costa et al., 2016; Gorostiaga et al., 2019; Van der Giessen et al., 2014), suggesting a bidirectional relation between these parental behaviors and adolescent depression. On one hand, a lack of autonomy support and high levels of psychological can precede and co‐occur with adolescent depression, because of the relevance of these parenting behaviors in adolescents' regulation of thoughts and feelings. Parental autonomy support can help adolescents to explore and deal with their own (negative) thoughts and feelings in (a trusting, supportive) relation with significant others in their life (Deci & Ryan, 2000). With negative thoughts and feelings at the center of depression, parental autonomy support may thereby be an important buffer. Contrary to autonomy support, parental psychological control has been considered a negative form of control that dysregulates adolescents' thoughts and feelings, diminishes their ability to establish emotional links with others, and to develop autonomous functioning and positive self‐views (Barber, 1996; Ryan & Deci, 2000). On the other hand, a lack of autonomy support and high levels of psychological control may co‐occur with and follow from adolescent depression, because of the manifestation of the disorder. Given the adolescent's negative thoughts and feelings and trouble with daily life, parents may be inclined to take over; to be overprotective towards their child in a controlling manner, thereby (unintentionally) communicating towards the adolescent they are lacking in competence for self‐care and undermining in opportunities to develop healthy regulatory strategies (Elzinga et al., 2022; Vigdal & Brønnick, 2022).

So far, however, research on the relations of parental autonomy support and psychological control with adolescent depression is mainly based on adolescent reports of these constructs, and there are no studies yet that examined the link in a clinical sample. Adolescents with a clinical diagnosis of depression possibly experience their parents' behavior more negatively due to their own negative beliefs and may be less likely to recognize and benefit from support from others, and more likely to expect and receive rejection (Coyne, 1976; Hale et al., 2008; Platt et al., 2017; Roth & Assor, 2012). In the current study, we aim to disentangle observed and adolescent‐perceived parenting behaviors in families with an adolescent with a current clinical depression as compared to HC families. This is of crucial importance given that perception and memory biases are well‐known characteristics of depression (Everaert & Koster, 2020; Platt et al., 2017). Lastly, we will test whether the affective state of adolescents with depression is more strongly influenced by their parents' behavior (observed autonomy support and psychological control). By examining these questions, we ultimately aim to gain insights for clinicians treating adolescents with depression.

### Current study

The current study has two overall aims. The first overall aim is to develop a reliable coding system for parental autonomy support (AS) and psychological control (PC) in different contexts of parent‐adolescent interactions, and gain insights into its ecological validity in a healthy control sample (HC families). More specifically, we will test the following hypotheses. In the more challenging problem solving task (vs. event planning and reminiscence), we expect observed parental AS to be lower and PC higher (1.1), and adolescents to report less parental listening/understanding (L/U) and more criticism/dominance (C/D) (1.2). We will explore whether observed parental AS and PC relate to adolescent‐perceived parental L/U and C/D and to adolescent affective state post‐task (1.3), and to adolescent‐perceived parental L/U and C/D in daily life (1.4).

The second overall aim is to disentangle observed and adolescent‐perceived parenting behavior in relation to adolescent depression. More specifically we will test the following hypotheses: In families with an adolescent with a clinical depression (vs. HC), we expect observed parental AS to be lower and PC higher (2.1), and adolescent‐reported parental L/U to be lower and C/D higher (2.2). Lastly, we will explore whether the relation of observed AS and PC with adolescent‐perceived L/U and C/D, and with adolescent affective state is different for adolescents with depression (vs. HC) (2.3). Preregistered hypotheses can be found via https://osf.io/rcbqz/?view_only=646785272d2742baaf11e64d57a9a474.

## METHODS

### Participants

The current study used data of the Dutch multi‐method, multi‐informant project ‘Relations and Emotions in Parent‐Adolescent Interaction Research’ (RE‐PAIR), in which the bidirectional relation between parent‐adolescent interactions and adolescent depression is researched. A group of families (data collected between June 2018 and December 2019) with an adolescent without psychopathology (i.e., healthy controls, HC; *N* = 80) and their parents (*N* = 148), and a group of families (data collected between June 2018 and March 2022) with an adolescent with a current Major Depressive Disorder (MDD) or dysthymia (adolescents with depression; *N* = 35) and their parents (*N* = 62) were included in the RE‐PAIR project. Sample characteristics are presented in Table 1. Of the adolescents with depression, 80.0% (*n* = 28) had a current MDD and 20.0% (*n* = 7) current dysthymia. Over half of the adolescents with depression had one or more comorbid disorder(s) (*n* = 22, 62.9%) with anxiety disorders being the most common (*n* = 19, 54.3%). Other comorbid disorders were attention deficit hyperactivity disorder, obsessive‐compulsive disorder, and conduct disorder. Data of 192 out of the 210 parent‐adolescent dyads (91.4%) were complete on all main variables (Supplementary Methods).

**TABLE 1:** Sample characteristics.

	HC families	DEP families	Group difference
Adolescents	(*N* = 80)	(*N* = 35)	
Biological sex, *n* (%) female	51 (63.7)	27 (77.1)	*χ* ^2^(1) = 2.00, *p* = .157
Age (years), *M* (SD)	15.90 (1.35)	15.60 (1.55)	*t*(57.65) = 1.00, *p* = .320
Highest level of education, *n* (%)			*χ* ^2^(5) = 4.01, *p* = .548
Lower vocational (Dutch: VMBO)	10 (12.5)	6 (17.1)	
Higher vocational (Dutch: HAVO)	20 (25.0)	6 (17.1)	
Pre‐university (Dutch: VWO)	33 (41.3)	13 (37.1)	
Secondary vocational (Dutch: MBO)	5 (6.3)	5 (14.3)	
Higher professional (Dutch: HBO)	2 (2.5)	2 (5.7)	
Other	10 (12.5)	3 (8.6)	
Ethnicity, *n* (%) White	73 (91.3)	23 (65.7)	*χ* ^2^(1) = 11.51, *p* = .001
PHQ‐9, *M* (SD)	4.88 (2.94)	19.97 (4.71)	*t*(45.99) = −17.54, *p* < .001
PBI, *M* (SD)			
Maternal care	31.91 (4.18)	27.06 (6.58)	*t*(46.58) = 4.02, *p* < .001
Maternal overprotection	3.59 (2.43)	6.11 (3.73)	*t*(47.26) = −3.66, *p* = .001
Maternal autonomy granting	14.33 (2.89)	13.23 (4.33)	*t*(47.96) = 1.38, *p* = .18
Paternal care	29.75 (5.19)	25.81 (6.34)	*t*(93) = 3.13, *p* = .002
Paternal overprotection	3.18 (2.35)	4.59 (2.66)	*t*(93) = −2.55, *p* = .012
Paternal autonomy granting	14.43 (2.47)	13.63 (3.69)	*t*(93) = 1.22, *p* = .224
Parents	(*N* = 148)	(*N* = 62)	
Biological sex, *n* (%) female	79 (53.4)	35 (56.5)	*χ* ^2^(1) = 0.17, *p* = .683
Age (years), *M* (SD)	49.00 (5.92)	50.13 (5.59)	*t*(208) = −1.28, *p* = .203
Highest level of education, *n* (%)			*χ* ^2^(2) = 4.97, *p* = .083
High school	16 (10.8)	14 (22.6)	
Secondary vocational (Dutch: MBO)	34 (23.0)	13 (21.0)	
Higher vocational education or university (Dutch: HBO, WO)	98 (66.2)	35 (56.5)	
Ethnicity, *n* (%) White	144 (97.3)	49 (79.0)	*χ* ^2^(1) = 19.59, *p* < .001
PHQ‐9, *M* (SD)	3.00 (3.56)	4.92 (5.07)	*t*(87.32) = −2.71, *p* = .008
MINI, *n* (%)			
Current psychopathology	23 (15.5)	22 (35.5)	*χ* ^2^(1) = 10.32, *p* = .001
Past psychopathology	45 (30.4)	32 (51.6)	*χ* ^2^(1) = 8.46, *p* = .004
PBI^a^, *M* (SD)			
Maternal care	32.91 (3.22)	29.91 (4.56)	*t*(49.62) = 3.52, *p* = .001
Maternal overprotection	3.86 (2.62)	5.66 (3.09)	*t*(112) = −3.20, *p* = .002
Maternal autonomy granting	14.03 (2.45)	12.60 (2.74)	*t*(112) = 2.76, *p* = .007
Paternal care^b^	29.67 (4.02)	28.78 (3.65)	*t*(94) = 1.00, *p* = .320
Paternal overprotection^b^	4.04 (2.39)	5.11 (2.69)	*t*(94) = −1.90, *p* = .060
Paternal autonomy granting^b^	14.07 (2.61)	13.30 (2.33)	*t*(94) = 1.35, *p* = .181

*Note*: Statistics presented here do not account for family clusters. See Supplementary Methods for psychometric properties. Group differences were tested with independent *t*‐tests (results were reported depending on Levene's test for equality of variances) and Pearson *χ* ^2^‐test.

Abbreviations: DEP, families with adolescent with current MDD/dysthymia; HC, families with healthy control adolescent; MINI, Mini International Neuropsychiatric Interview; PBI, Parental Bonding Inventory; PHQ‐9, Patient Health Questionnaire‐9.

^a^ PBI concerns self‐reported parental bonding with participating child in the current study.

^b^ Missing data PBI of one father HC group (thus *n* = 68 for father‐report of PBI).

All adolescents were aged between 11 and 17 years at time of inclusion, willing to participate and living with at least one primary caregiver, and attending (or completed) high school or higher education. Adolescents as well as their parent(s) were required to have a sufficient command of the Dutch language. HC families were excluded if the adolescent had any psychopathology currently or in the past 2 years, had a lifetime depressive disorder, a history of psychological treatment, or used medication for psychological disorders or sleep medication. Families with an adolescent with depression were included if the adolescent had a current primary diagnosis of MDD or dysthymia. Families in this group were excluded if the adolescent used unstable doses of antidepressants, if safety could not be ensured because of suicidal tendencies (suicidal ideation per se was no exclusion criterion) or severe auto‐mutilation, or in case of current comorbid intellectual disability, psychosis, eating disorders, substance use disorders, and autism spectrum disorders. The Kiddie‐Schedule for Affective Disorders and Schizophrenia – Present and Lifetime (K‐SADS‐PL; Reichart et al., 2000; Supplementary Methods) was used to verify in‐ and exclusion criteria in both subsamples.

### Procedure

Healthy control families were recruited via (social) media, advertisements, and flyers. Families with an adolescent with depression were recruited via advertisements, and in collaboration with mental health care facilities in the area of Leiden, the Netherlands. Families interested in participation were informed and screened (i.e., brief check of in‐ and exclusion criteria) by phone. For families with an adolescent with depression an appointment was made to diagnostically interview the adolescent using the K‐SADS‐PL (Reichart et al., 2000), to check further in‐ and exclusion criteria. Subsequently (after screening for HC families, and after the K‐SADS‐PL interview for families with an adolescent with depression) participation consisted of four study parts: online questionnaires, one research day at the laboratory (during which the K‐SADS‐PL was assessed for HC adolescents), 14 consecutive days of EMA, and an fMRI scan session. All travel expenses were compensated. Adolescents received 15–55 euros and parents 73–103 euros of monetary compensation, depending on the study parts they participated in. Vouchers of 75 euros were raffled based on EMA compliance of the families. Lastly, adolescents with depression received written feedback based on their own report of several questionnaires (administered prior and during the research day). In the current study, we used part of the data of the lab assessment and the EMA.

The RE‐PAIR study was approved in May 2018 by the Medical Ethical Committee of the Leiden University Medical Centre (LUMC; NL62502.058.17) and conducted in accordance with the declaration of Helsinki and the Dutch Medical Research Involving Human Subjects Act (WMO). Participants signed informed consent, and both parents with legal custody signed additional informed consent in case their child was younger than 16 years.

#### Parent–child interaction tasks in the lab

Parent‐adolescent dyads completed three videotaped interaction tasks. The adolescent participated with both parents separately, in counterbalanced order. The researcher introduced the tasks one at a time, turned an hourglass to indicate the start of each task and the lapse of time, left the observation room, and came back after the indicated time had passed. Directly after each interaction task, the dyad filled out several questions about parenting behavior during the task and their own affect. The three interaction tasks were:

- Problem solving interaction task (10 min; Davis et al., 2000). At the start of the lab assessment, the adolescent (once about mother, once about father) and parent(s) independently completed an adapted version of the Issues Checklist (Robin & Weis, 1980). This checklist contains an overview of topics that are commonly of issue to parent‐adolescent dyads and has an open space to add topics. The participants indicated on a 5‐point scale the frequency (1 = *never*, 5 = *very often*) and the intensity (1 = *calm*, 5 = *very intense*) of arguing over each topic in the past 4 weeks. The researcher selected the three topics that were reported to occur most frequently and intensely by the dyad, and wrote them on three numbered pieces of paper. If the adolescent and parent were inconsistent in their report of the topics, the parent's report was leading. The dyad was asked to discuss the topic(s) by elaborating on their point of view, and trying to find a solution to the issue. When they finished discussing the first topic and there was time left, they could proceed with the second and third topic.
- Event planning interaction task (6 min; adapted version of task by Schwartz et al., 2012). The dyad was asked to plan a (weekend) trip they would both enjoy, with unlimited budget. They were suggested to discuss their transport, activities, lunch/dinner plans, et cetera. When they finished and there was time left, they could proceed planning a second trip.
- Reminiscence interaction task (6 min; adapted version of task by Sheeber et al., 2012). At the start of the lab visit, the adolescent wrote down two emotional events they had experienced that made them feel sad, bad, or disappointed, and indicated how intense these events were on a 3‐point scale (somewhat, moderately, very). This concerned events the parent was not involved in and, preferably, had not yet heard of. The adolescent was informed beforehand that they would be discussing the event(s) with their parent(s) during the interaction task. The adolescent was instructed to share the emotional event(s) with their parent and to start with the most intense one.

### Measures

#### Observed parental autonomy support and psychological control in lab setting

A new coding system, Coding Parental Autonomy Support and Psychological Control in Adolescence (CASPCA), was developed (coding manual included in Supplementary Methods) and used to (macro)code parental AS and PC behaviors per parent‐adolescent interaction task. The CASPCA was developed based on behaviors as described in three existing coding systems (Barber, 1996; Hauser Kunz & Grych, 2013; Wuyts et al., 2018) and a questionnaire (Mageau et al., 2016) (Table S1), and on initial observations of videos of the current dataset. Previous coding systems included very relevant behaviors, but we felt the need to develop a new coding system for three main reasons. First, we aimed to use a coding system that includes AS and PC, but treats these as separate constructs. Barber (1996) described PC behaviors and Wuyts et al. (2018) AS behaviors (vs. controlling), thereby focusing on one construct. Second, we aimed to examine autonomy support rather than autonomy granting. Thereby including parents' attempts to empathize with and more deeply understand their child following their child's expressions, in addition to parents' attempts to stimulate their child to initiate expressions. Hauser Kunz and Grych (2013) developed a coding system including autonomy granting and PC as separate constructs. And lastly, we aimed to use a concise number of subscales, covering the relevant behaviors of AS and PC. Previous coding systems included very relevant behaviors, but we wanted to further group these together. Table S1 presents the behaviors of existing coding systems and the questionnaire that were used to develop the CASPCA subscales. We decided not to include ‘love withdrawal’ in the coding of PC, because we expected this behavior would not occur during instructed parent‐adolescent interactions in the lab setting. Lastly, we used the format of the negativity scale of the coding system by Allen et al. (2001), as a format to code intensities of PC behaviors.

Parental AS was coded on three 9‐point subscales: (1) encouragement of the adolescent's input (i.e., (non)verbal encouragement, demonstrating patience); (2) explanation of the parent's own motivations (i.e., clear/calm/respectful manner, adjusted to adolescent's mood and understanding); (3) receptiveness to the adolescent's input (i.e., active acceptance, relating and understanding). A higher score indicates higher levels of the behavior (see coding manual in Supplementary Methods). The subscale ‘Explaining motivations’ was only coded for the problem solving task; giving motivations in itself was not considered to be autonomy supportive in the event planning (can deduce positivity of interaction) and reminiscence (indicates focus on own perspective rather than adolescent's perspective) task. To ensure that the (absence of) relations of observed AS with other variables was not due to including ‘Explaining motivations’ only for the problem solving task, we also computed a mean score for this task based on ‘Encouraging input’ and ‘Receptiveness to input’ only.

Parental PC was also coded on three 9‐point subscales: (1) constraining the adolescent's expressions (i.e., dominating behavior, dominating content, disinterest); (2) guilt induction (i.e., making adolescent unreasonably responsible, prioritizing own perspective); (3) invalidating the adolescent's emotions (i.e., assigning values, minimalizing). A higher score indicates higher levels of the behavior (see coding manual in Supplementary Methods). The mean of subscales was used to respectively indicate the overall level of AS and PC. Thus, six scores were assigned to each parent (three interaction tasks * two constructs).

Two groups of undergraduate students in psychology and family studies (group 1: *n* = 6, group 2: *n* = 7) were trained in five sessions and reliably coded a reliability set of 30 videos (average measures ICC AS *r* = .96, PC *r* = .94). The first session of the training consisted of an introduction to the constructs, coding system and some example fragments of the behaviors. Next, the students independently coded three to six videos in preparation per session (all videos of exclusion families), which were discussed during the subsequent sessions. Coders were blind to group status of the participants and other outcome variables (e.g., perceived parenting behaviors). The first group of students coded all videos that were available at that point (*n* = 148 HC parents, *n* = 30 parents of adolescents with depression). The second group (*n* = 7) double‐coded the videos (three tasks) of 20 parents of the HC families (average measures ICC AS *r* = .73, PC *r* = .59) to ensure they also coded a mix of the subsamples and coded the videos of the remaining 32 parents of adolescents with depression. For the double‐coded videos, the second round of coding was used as the final scores.

#### Adolescent‐perceived parental listening/understanding and criticism/dominance

Adolescent‐perceived parental L/U and C/D were assessed directly after each interaction task. Adolescents reported on four items (i.e., “How well did your [mother/father] [listen to/understand] you?” and “How [critical/dominant] was your [mother/father] towards you?”) on a 7‐point scale (1 = *not at all*, 7 = *very*). The means of the items on listening and understanding, and of the items on criticism and dominance were used to respectively indicate the level of adolescent‐perceived L/U and C/D. A higher score represents higher levels of the specific behavior. Thus, six scores were assigned to each parent–child dyad (three interaction tasks * two constructs).

#### Adolescent positive and negative affect

Adolescent positive and negative affect were assessed prior to the start of the first interaction task (baseline) and directly after each interaction task using an adapted and shortened version of the Positive and Negative Affect Schedule for Children (PANAS‐C; Ebesutani et al., 2012; Watson et al., 1988). The adolescent reported on four items (i.e., “How [happy/sad/relaxed/irritated] are you feeling at the moment?”) on a 7‐point scale (1 = *not at all*, 7 = *very*). The mean of the items on happy and relaxed, and of the items on sad and irritated respectively indicated the level of adolescent positive and negative affect. Higher scores represented higher levels of positive and negative affect. In the main analyses, pre‐task affect per task concerned affect prior to the start of the specific interaction task. Thus, baseline affect is the pre‐task affect for the problem solving task; post‐problem solving affect is the pre‐task affect for the event planning task; and post‐event planning affect is the pre‐task affect for the reminiscence task. A total of 12 scores were assigned to each parent–child dyad for mean levels of adolescent positive and negative affect in the lab (three pre‐task * two constructs + three post‐task * two constructs).

#### Adolescent‐perceived parental listening/understanding and criticism/dominance in daily life

For a detailed description of the EMA procedure, see (Janssen et al., 2021). We preregistered to use proximity‐triggered questionnaires, but because we had less data available for these measures, we decided to use the fixed daily questionnaires. Families filled in the EMA after the lab assessment. Adolescents received four fixed questionnaires per day, for 14 consecutive days, in which they were asked whether they had interacted with their parent(s) since the last questionnaire. If the adolescent had interacted face‐to‐face with one or both parents who had also participated in the lab assessment of RE‐PAIR, they reported on four items (i.e., “How well did your [mother/father] [listen to/understand] you?” and “How [critical/dominant] was your [mother/father] towards you?”) on a 7‐point scale (1 = *not at all*, 7 = *very*) per parent. We excluded all interactions via phone and online interactions. Two scores were assigned to each parent–child dyad: the mean of listening and understanding of all questionnaires across all days indicated the level of adolescent‐perceived L/U in daily life and the mean of criticism and dominance indicated the level of adolescent‐perceived C/D. A higher score represents higher levels of the specific behavior throughout the EMA period. HC adolescents completed 11.24 questionnaires on average (SD = 7.14, range [0, 33]).

### Statistical analyses

Contrary to our preregistration, we used the lme4 package (Bates et al., 2015) for multilevel modeling in R software (version 4.3.0; R Core Team, 2023) and the permute package (Simpson et al., 2022) for permutation testing to correct for multiple testing. Multilevel modeling was used because of the nested structure of our data: Standardized (*z*‐scores) observations (level 1) are clustered within persons (level 2), which are clustered within families (level 3). Parameters are added step‐by‐step, and model fit improvement is tested with the Likelihood ratio test. Parameters of main interest per model are kept in the model regardless of the significance of model fit improvement. In all models, biological sex (male, female) of parent and child, and child age are included as covariates in the final step.

We performed permutation tests to correct for multiple testing per combination of outcomes (because observed and perceived parenting as well as affect were split in a positive and negative component, doubling all models) in case of significant results. The data are permuted with a thousand shuffles and the observed coefficients of the models (*t* value) are compared to the permuted coefficients of the models, in order to estimate the robustness of the observed effect (Dudoit et al., 2003). This comparison was always based on the simplest model, including only the fixed effects of the independent variable and the type of task.

## RESULTS

### Descriptive analyses

Healthy control families and families with an adolescent with depression did not differ on most of the demographic variables: adolescents' age, biological sex, and level of education, and parents' age, biological sex, and level of education (all *p'*s > .05; Table 1). HC adolescents and parents reported a white ethnicity more often than adolescents with depression and their parents (*p*'s < .05; Table 1). Across tasks and groups, we found moderate negative correlations between AS and PC (*r* = −.63, *p* < .001), adolescent‐perceived L/U and C/D (*r* = −.50, *p* < .001), adolescent positive and negative affect (across tasks: *r* = −.51, *p* < .001), and adolescent‐perceived L/U and C/D in daily life (*r* = −.62, *p* < .001). Correlations per task per group are presented in Table S2. Descriptive statistics of observed and adolescent‐perceived parenting behaviors and of adolescent affect are presented in Table 2.

**TABLE 2:** Descriptive statistics main variables.

	HC families				DEP families			
	*n*_parents_	*n*_adolescents_	*M* (SD)	*α*	*n*_parents_	*n*_adolescents_	*M* (SD)	*α*
Observed parental autonomy support								
Problem solving	148	80	5.77 (1.56)	.83	62	35	5.60 (1.70)	.87
Event planning	148	80	6.17 (1.47)	.78	62	35	5.84 (1.67)	.75
Reminiscence	146	79	6.60 (1.47)	.76	62	35	6.26 (1.79)	.89
Observed parental psychological control								
Problem solving	148	80	2.98 (1.35)	.67	62	35	2.95 (1.44)	.68
Event planning	148	80	2.35 (0.97)	.43	62	35	2.53 (1.13)	.51
Reminiscence	146	79	2.40 (1.06)	.42	62	35	2.56 (1.42)	.65
Adolescent‐perceived parental listening/understanding								
Problem solving	148	80	5.83 (1.12)	.86	61	34	5.34 (1.55)	.94
Event planning	146	80	6.37 (0.80)	.71	62	35	6.01 (1.16)	.92
Reminiscence	143	79	6.36 (0.78)	.76	62	35	5.83 (1.16)	.88
Adolescent‐perceived parental criticism/dominance								
Problem solving	148	80	2.81 (1.20)	.56	61	34	2.93 (1.40)	.64
Event planning	146	80	1.83 (0.96)	.37	62	35	2.06 (1.27)	.81
Reminiscence	143	79	1.81 (1.01)	.61	62	35	2.29 (1.37)	.81
Adolescent positive affect								
Baseline	147	80	5.10 (1.04)	.68	62	35	3.60 (1.17)	.74
Problem solving	148	80	5.32 (0.88)	.62	62	35	3.66 (1.30)	.75
Event planning	146	80	5.82 (0.83)	.58	62	35	4.43 (1.30)	.78
Reminiscence	143	79	5.15 (1.12)	.74	62	35	3.72 (1.26)	.83
Adolescent negative affect								
Baseline	147	80	1.43 (0.77)	.53	62	35	2.69 (1.20)	.64
Problem solving	148	80	1.38 (0.64)	.46	62	35	2.91 (1.41)	.64
Event planning	146	80	1.15 (0.36)	.46	62	35	2.34 (1.29)	.60
Reminiscence	143	79	1.55 (0.80)	.37	62	35	2.89 (1.38)	.65
Adolescent‐perceived parenting daily life (ecological momentary assessment)								
Parental list./und.	142	79	5.63 (0.96)	.90	58	34	5.39 (0.97)	.90
Parental cr./dom.	142	79	1.88 (0.95)	.77	58	34	2.09 (1.00)	.84

*Note*: Statistics presented here do not account for family clusters. HC = families with healthy control adolescents; DEP = families with adolescents with current MDD/dysthymia. *α* indicates the Cronbach's alpha for internal reliability of the subscales per measure.

### Multilevel analyses

**FIGURE 1:**
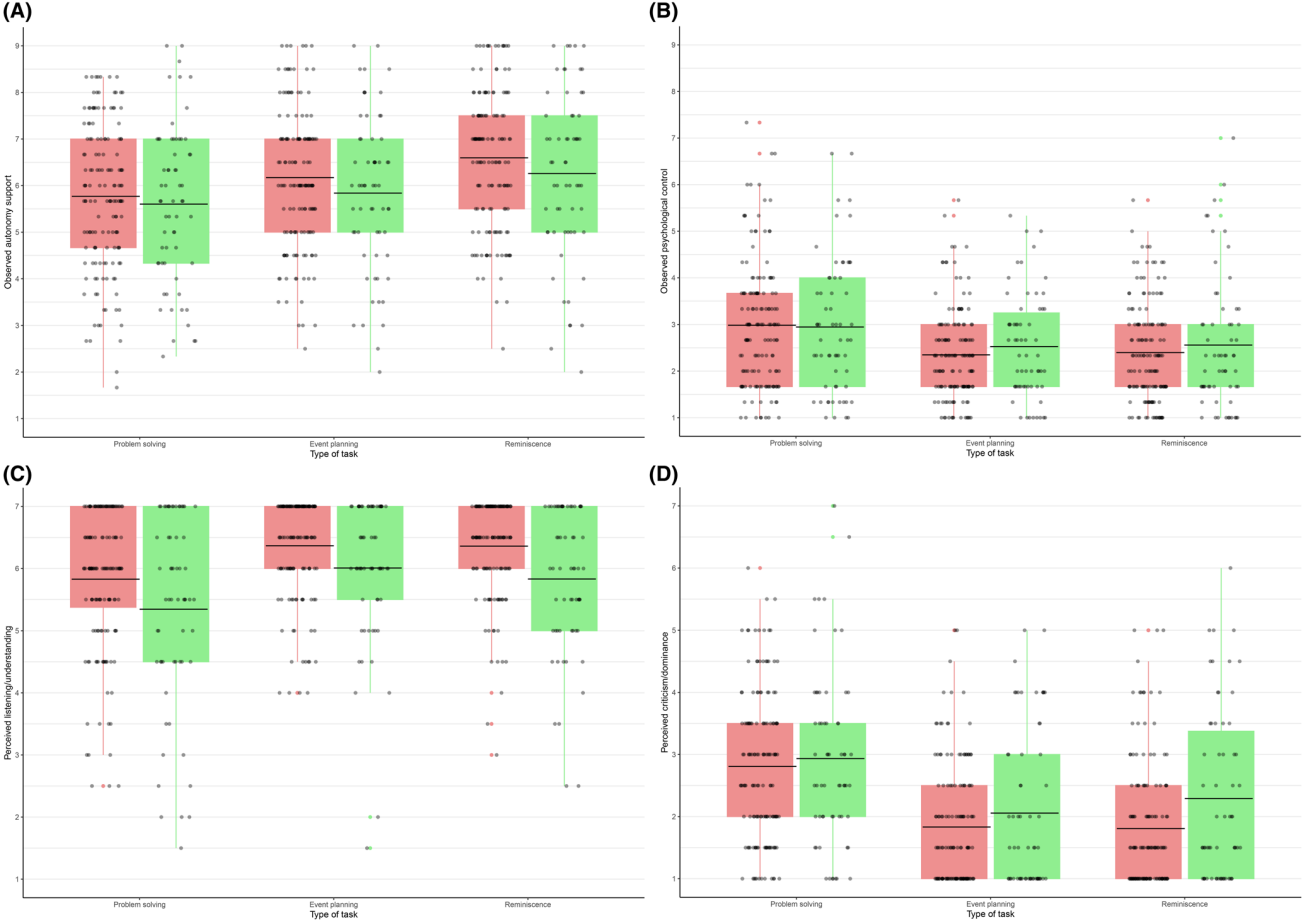
Distribution of observed and adolescent‐perceived parenting behaviors per group per task. *Note*: Distributions presented here do not account for family clusters. HC = families with a healthy control adolescent; DEP = families with an adolescent with current MDD/dysthymia. Sample sizes per type of task and type of parenting behavior are reported in Table 2. Bar in the boxplot indicates mean level.

Separate models were run for observed AS and PC because of moderate correlations between these variables, rather than including them as multiple predictors in one model. All model fit statistics and final models (in bold) are presented in Table S3 (aim one) and Table S8 (aim two). Intraclass correlations indicating the proportion variance accounted for by the person and family level are presented per final model in Tables S4–S7 (aim one) and Tables S9–S11 (aim two). Distributions of observed and adolescent‐perceived parenting behaviors are presented in Figure 1.

#### Aim one: Coding AS and PC in HC sample

##### Task differences in observed parenting behaviors (H1.1)

We first tested whether parents showed less AS and more PC in the problem solving than event planning and reminiscence task (H1.1). In line with our hypotheses, we found significantly lower levels of observed AS in the problem solving than event planning (*B* = 0.262, SE = .099, *p* = .009) and reminiscence task (*B* = 0.541, SE = .099, *p* < .001). Observed AS was significantly higher in the reminiscence than event planning task (*B* = 0.279, SE = .099, *p* = .005). We furthermore found significantly higher levels of observed PC in the problem solving than event planning (*B* = −0.543, SE = .103, *p* < .001) and reminiscence task (*B* = −0.504, SE = .103, *p* < .001), but no significant difference in observed PC between the reminiscence and event planning task (*B* = 0.039, SE = .103, *p* = .085). Results are presented in Table S4.

The permutation test (based on model including fixed effects of type of task) showed that the differences in observed AS in the problem solving than event planning task, and in observed AS in the reminiscence than event planning task were almost fully robust: Respectively 4.0% and 1.5% of permuted coefficients were larger than the observed coefficient. The other significant effects were fully robust to the correction.

##### Task differences in adolescent‐perceived parenting behaviors (H1.2)

We tested whether adolescents perceived their parents as less L/U and more C/D during the problem solving than event planning and reminiscence task (H1.2). The model had trouble converging when including all covariates due to its complexity, maximizing iterations did not solve the problem. We only included the significant covariate (parental biological sex) to ensure a stable model. In line with the hypothesis, adolescent‐perceived L/U was significantly lower in the problem solving than event planning (*B* = 0.566, SE = .075, *p* < .001) and reminiscence task (*B* = 0.578, SE = .072, *p* < .001). There was no significant difference in adolescent‐perceived L/U between the reminiscence and event planning task (*B* = 0.012, SE = .076, *p* = .873). In line with the hypothesis, adolescent‐perceived C/D was higher in the problem solving than event planning (*B* = −0.836, SE = .074, *p* < .001) and reminiscence task (*B* = −0.885, SE = .075, *p* < .001). There was no significant difference in adolescent‐perceived C/D between the reminiscence and event planning task (*B* = −0.049, SE = .075, *p* = .513). Results are presented in Table S4. The permutation test (based on model including fixed effects of type of task) showed that the significant differences were fully robust.

##### Observed parenting behaviors' relation with adolescent‐perceived parenting behaviors and adolescent affect (H1.3)

###### Adolescent‐perceived parenting behaviors

We tested whether observed AS and PC related to adolescent‐perceived L/U and C/D (H1.3). Observed AS was not significantly related to adolescent‐perceived L/U (*B* = 0.072, SE = .039, *p* = .067) when controlling for type of task and the covariates. Higher levels of observed AS significantly related to lower levels of adolescent‐perceived C/D (*B* = −0.120, SE = .038, *p* = .002), while controlling for type of task and the covariates. Higher levels of observed PC related to lower levels of adolescent‐perceived L/U (*B* = −0.093, SE = .038, *p* = .014) and higher levels of adolescent‐perceived C/D (*B* = 0.107, SE = .037, *p* = .004), while controlling for type of task and the covariates. Results are presented in Table S5.

The permutation test (based on model including fixed effects observed behavior and type of task) showed that the relation between observed AS and adolescent‐perceived C/D, and between observed PC and adolescent‐perceived L/U and C/D were robust: Respectively 0.6%, 6.6%, and 2.3% of the permuted coefficients were larger than the observed coefficients.

###### Adolescent affect

We tested whether observed AS and PC related to adolescents positive and negative affect (H1.3). Pre‐task positive and negative affect was respectively added per set of multilevel modeling. Observed AS was not significantly related to adolescent positive (*B* = 0.054, SE = .060, *p* = .368) and negative (*B* = −0.020, SE = .041, *p* = .629) affect. However, there was a significant interaction effect between observed AS and type of task on adolescent positive affect. Specifically, during the reminiscence task, higher levels of observed parental AS related to *lower* levels of adolescent positive affect, while controlling for pre‐task affect (reminiscence vs. problem solving; *B* = −0.224, SE = .082, *p* = .006; reminiscence vs. event planning; *B* = −0.216, SE = .084, *p* = .011). Observed PC was not significantly related to adolescent positive (*B* = 0.036, SE = .046, *p* = .440) and negative (*B* = −0.008, SE = .040, *p* = .834) affect. Results are presented in Table S6.

##### Observed parenting behaviors' relation with adolescent‐perceived parenting behaviors in daily life (H1.4)

We tested whether observed AS and PC (mean levels across the three interaction tasks) related to adolescent‐perceived L/U and C/D in daily life (H1.4). The person level did not vary and a cluster at that level (and relating ICC analysis) was not applicable. Observed AS did not relate to adolescent‐perceived L/U (*B* = 0.114, SE = .076, *p* = .136) and C/D (*B* = −0.051, SE = .078, *p* = .517) in daily life. Observed PC did significantly relate to adolescent‐perceived L/U in daily life (*B* = −0.176, SE = .068, *p* = .011), with higher levels of observed PC in the lab relating to lower levels of adolescent‐perceived L/U in daily life. Observed PC did not relate to adolescent‐perceived C/D in daily life (*B* = 0.081, SE = .073, *p* = .266). Results are presented in Table S7.

The permutation test (based on model including fixed effects observed behavior) showed that the relation between observed PC and adolescent‐perceived L/U in daily life was robust: Only 6.6% of the permuted coefficients were larger than the observed coefficient.

We checked whether the results changed when excluding ‘Explaining motivations’ as a subscale in the mean score of AS in the problem solving task (see Measures). This was not the case, the (in)significance of none of the effects changed.

#### Aim two: Comparing families with an adolescent with depression to HC families

##### Observed parenting behaviors (H2.1)

We tested whether parents of adolescents with depression (vs. HC) showed less AS and more PC (H2.1). There was no significant group effect for observed AS (*B* = −0.184, SE = .117, *p* = .120), nor for observed PC (*B* = 0.083, SE = .100, *p* = .410) (Table S9), the hypothesis was therefore not confirmed. Additionally, we explored whether there were group differences in the three subscales of AS and the three of PC, which was not the case (all *p*'s > .10).

##### Adolescent‐perceived parenting behaviors (H2.2)

We tested whether adolescents with depression (vs. HC) perceived their parents as less L/U and more C/D (H2.2). In testing the group effect on adolescent‐perceived L/U, the model had trouble converging when including the random slopes. Maximizing iterations did not solve the problem and the parameter was dropped to ensure a stable model. In line with the hypothesis, adolescents with depression perceived their parents as significantly less L/U than the HC adolescents (*B* = −0.431, SE = .145, *p* = .004). In testing the group effect on adolescent‐perceived C/D, the interaction term (group*type of task; model 5) did not significantly improve model fit, but showed a trend (*p* = .067). To understand the (possible) effect at play, the parameter was therefore included. There was no significant main effect of group on adolescent‐perceived C/D (*B* = 0.136, SE = .163, *p* = .406), but there was a significant interaction effect of group by type of task (reminiscence vs. problem solving: *B* = 0.293, SE = .133, *p* = .028), indicating that adolescents with depression (vs. HC) reported higher levels of C/D specifically in the reminiscence task. Results are presented in Table S9.

The permutation test (based on model including fixed effect of group and type of task) showed that the group difference in adolescent‐perceived L/U was not robust: 53.5% of permuted coefficients were larger than the observed coefficient.

##### Adolescent views on parenting behaviors (H2.3)

We tested whether the relation of observed AS and PC with adolescent‐perceived L/U and C/D was different for adolescents with depression (vs. HC) (H2.3). There was no effect of group on the relation of observed AS with adolescent‐perceived L/U (*B* = 0.062, SE = .063, *p* = .322) and C/D (*B* = 0.079, SE = .065, *p* = .223), neither on the relation of observed PC with adolescent‐perceived L/U (*B* = −0.067, SE = .060, *p* = .264) and C/D (*B* = 0.011, SE = .062, *p* = .865). In both groups, observed AS negatively related to adolescent‐perceived C/D, and observed PC negatively related to adolescent‐perceived L/U and positively related to adolescent‐perceived C/D. Results are presented in Table S10.

##### Adolescent affective responses to parental observable behaviors (H2.3)

We tested whether the relation of observed AS and PC with adolescent positive and negative affect was different for adolescents with depression (vs. HC) (H2.3). Pre‐task positive and negative affect was respectively added per set of multilevel modeling. There was no effect of group on the relation between observed AS and adolescent positive (*B* = 0.029, SE = .054, *p* = .589) and negative (*B* = −0.037, SE = .052, *p* = .486) affect, nor for the relation between observed PC and adolescent positive affect (*B* = −0.067, SE = .050, *p* = .177). In both groups, observed AS did not relate to adolescent positive and negative affect, and observed PC did not relate to adolescent positive affect. However, group status did interact with the relation between observed PC and negative affect (*B* = 0.122, SE = .050, *p* = .015). Observed PC related to more adolescent negative affect in adolescents with depression, but not in HC adolescents. Results are presented in Table S11.

The permutation test (based on model including fixed interaction effect, pre‐task affect, and type of task) showed that the interaction effect of group status on the relation between observed PC and adolescent negative affect was almost fully robust: Only 0.7% of the permuted coefficients were larger than the observed coefficient.

We checked whether the results changed when excluding ‘Explaining motivations’ as a subscale in the mean score of AS in the problem solving task (see Measures). This was not the case, the (in)significance of none of the effects changed.

#### Covariates

We found significant effects for the covariates biological sex of the parent and pre‐task affect in aim one (Tables S4–S6) and two (Tables S9–S11). Adolescents perceived their fathers as more L/U and less C/D than their mothers, and positive and negative pre‐task affect respectively positively related to positive and negative post‐task affect. Further, in studying both aims, type of task as a variable of interest had a significant effect on adolescent affect. Positive affect was lower and negative affect higher in the problem solving than event planning and reminiscence, and in the reminiscence than event planning task. Specifically in aim one, HC boys perceived their parents as significantly less C/D than girls. Lastly, we additionally included ethnicity as a covariate in aim two (group comparisons), given the significant group difference on this variable (see Descriptive Analyses). The (in)significance of the effects reported in aim two did not change.

## DISCUSSION

The current study aimed to (1) develop a reliable coding system for parental autonomy support and psychological control in different contexts of parent‐adolescent interactions, gain insights into its ecological validity, and (2) disentangle observed and adolescent‐perceived parenting behaviors in relation to adolescent depression. First, we developed a new and reliable coding system for observed parental autonomy support and psychological control, that showed ecological validity. With regards to adolescent depression, adolescents with depression (vs. HC) perceived their parents as somewhat less listening/understanding across interactions (effect is not robust) and perceived them as more critical in emotional interactions, but their parents did not show less observable autonomy support nor more psychological control. And lastly, following observed parental psychological control the negative affective state of adolescents with depression (but not HC) worsened.

### Aim one: Coding observed parental autonomy support and psychological control

Results of the current study importantly add to the understanding of parental autonomy support and psychological control during adolescence. We reached our first aim by developing a new and reliable coding system, CASPCA, with separate assessments for observed parental autonomy support and psychological control, that is sensitive to the context of parent‐adolescent interactions. Aligning previous work (Barber et al., 2005; Hauser Kunz & Grych, 2013), the current study shows that it is important to not put autonomy support and psychological control on one continuum, because of moderate negative correlations (see Table S2) between these behaviors and the different patterns of results (e.g., psychological control, but not autonomy support, related to adolescent‐perceived listening/understanding in the lab and in daily life).

As expected, autonomy support was lower and psychological control higher in the more challenging (i.e., demanding, stressful) problem solving task than the event planning and reminiscence tasks, thereby aligning previous studies (Branger et al., 2019; Grolnick et al., 2002; McCurdy et al., 2020). The same effects (more negative in problem solving task) were found for adolescent‐perceived parental listening/understanding and criticism/dominance. Interestingly, parents showed even more autonomy support in the reminiscence than event planning task, and are thus better able to display this behavior in an emotional context. Adolescents did not perceive their parents as more listening/understanding in the reminiscence task, which may be explained by a ceiling effect of this behavior (*M* = 6.5 out of a 7‐pointscale in event planning task) or an increased focus of adolescents on the self, given the nature of the task (ding a personal emotional event). In conclusion, it is important to consider the context of the interaction in research (and clinical practice), because different contexts elicit different levels of observed autonomy support and psychological control, and of adolescent‐perceived listening/understanding and criticism/dominance.

The CASPCA coding system is ecologically valid in measuring parenting behaviors that are relevant to adolescents' experiences of feeling listened to/understood and feeling criticized/dominated by their parents. Observed autonomy support negatively related to adolescent‐perceived criticism/dominance, and observed psychological control negatively related to adolescent‐perceived listening/understanding and positively to criticism/dominance. Observed psychological control also related to adolescents' daily life experiences of feeling listened to and understood. Previous studies showed that more negative observed parenting relates to more negative *global* levels of adolescent‐perceived parenting (Wuyts et al., 2018), and that observed parenting behavior in the lab related to adolescents' perceptions of their parents' momentary behavior in daily life (i.e., expressed affect of parent towards child; Griffith et al., 2018). Together these and current findings suggest that observing parenting behaviors in a lab setting holds ecological validity to adolescents' experiences of momentary parenting behaviors (in daily life).

The results further indicate that, although autonomy support and psychological control should be treated as separate constructs, they are clearly related to each other and show cross‐over effects. A parent who shows higher levels of psychological control (e.g., forcing solutions, invalidating the adolescent) makes their child feel more criticized and dominated, but also less listened to and understood. Whereas a parent who shows low levels of autonomy support (e.g., little to no questions, patience, or genuine interest) does not directly make their child feel less listened to and understood, but it does make the child feel more criticized and dominated. Psychological control may have a more profound, or direct, effect on adolescents' experiences than autonomy support. Observed autonomy support and psychological control generally did not relate to HC adolescent affective state. However, in the reminiscence task, observed AS related to *lower* positive affect, while controlling for pre‐task affect. Parental expressions of AS in this emotional task notably related to adolescents feeling less well instead of better. Further research could examine the mechanism underlying this relation.

### Aim two: Parental autonomy support and psychological control in the context of adolescent depression

Using the newly developed coding system, we have also acquired important insights into observed and perceived parenting in the context of adolescent depression. Previous work showed that global levels of perceived parental autonomy support and psychological control relate to adolescent depressive symptoms, in mainly normative populations (Barber et al., 2005; Chyung et al., 2022; Gorostiaga et al., 2019; Van der Giessen et al., 2014). The current study did not find different levels of observed autonomy support and psychological control in a clinical (vs. HC) sample, whereas adolescents with depression did perceive their parents as less listening/understanding across the interaction tasks and as more critical/dominant in the emotional reminiscence task. Combining current and previous results, research shows that perspectives on parenting matter, and that adolescent depression appears to relate to perceived, but not observed, parenting. Previous findings on overall perceived autonomy support and psychological control can thus be translated to perceived listening/understanding and criticism/dominance in specific interactions between parents and adolescents with a clinical depression. These behaviors cover important parts of respectively autonomy support and psychological control, but are not equivalent to each other. Possibly, different nuances in these constructs may explain the divergent results. Another important possible explanation for the divergent results may be that adolescents with depression (vs. HC) have more negative perceptions. This aligns meta‐analytic evidence showing stronger effects for child‐reports than observations of parenting on childhood depression (McLeod et al., 2007; Pinquart, 2017). It should be noted that the group difference in perceived listening/understanding was clearly significant (*p* = .004), but not robust to the correction (53.5% of the imputed coefficients were larger than the observed coefficient). This may indicate heterogeneity in the sample, with more negative perceptions in some adolescents with depression, but not others; further research is needed.

Several factors may explain the more negative perceptions (relative to observations) of adolescents with depression. First, previous experiences of parenting (e.g., more negative in daily life than in lab setting) or the overall parent–child bond may have shaped the negative perception bias of adolescents with depression in these specific interactions. The adolescents' representation of their parents' behavior may be more negative, thereby affecting their reports of parenting in specific interactions. Second, the adolescent's overall affective state may have led to a negativity bias. Van der Kaap‐Deeder et al. (2023) found that adolescents' affect preceded their perceptions of parenting in daily life. Similarly in our study, adolescents' baseline affective state (reported in Table 2) may have influenced perceived parenting behaviors. A last possible factor is that expressions of parents' negative behavior in the interactions, are perceived more negatively by adolescents with depression (vs. HC). However, current results do not confirm this: The relation of observed autonomy support and psychological control with adolescent‐perceived listening/understanding and criticism/dominance did not differ between groups.

Lastly, the current study highlights the negativity of observed parental psychological control in the context of adolescent depression, even though parents of adolescents with depression did not express more of this behavior when interacting with their child (i.e., no group differences in observed psychological control). Adolescents with depression reported more negative affect following their parents' psychological control. Interestingly, this was not the case for HC adolescents. This is in line with previous self‐report studies that indicate that psychological control dysregulates adolescents' feelings (Barber, 1996; Deci & Ryan, 2000), specifically in adolescents who already have difficulties in sadness regulation (Cui et al., 2014). The latter result is hereby extended to the relation of observed psychological control with adolescent affective state in interactions with their parents. Psychological control can be considered as a form of rejection towards the child's thoughts, feelings, and cognitions, and depression is known to relate to higher rejection sensitivity (e.g., Gao et al., 2017; Garber et al., 1997). The current study shows that the negative affective state of adolescents with depression worsened after their parents' expressed psychological control, suggesting that these adolescents are indeed sensitive to this rejecting type of parenting behavior. Remarkably, HC adolescents appear to be resilient to this parenting behavior in specific interactions with their parents. The feeling of ‘walking on eggshells’ (i.e., carefully considering own behavior to prevent the child from feeling worse) that parents of adolescents with depression reported in a qualitative study (Stapley et al., 2016) thereby seems to be confirmed by data in our study; negative parenting behavior in specific interactions indeed makes the adolescent (with depression) feel worse.

### Clinical implications

Results of the current study highlight the importance to consider multiple perspectives on parent‐adolescent interactions in the clinical setting and can be translated into three main implications. First, our study shows that different contexts elicit different levels of specific parenting behaviors. A stressful, demanding context (i.e., problem solving) elicits more negative parenting behaviors than a more positive (i.e., event planning) and emotional (i.e., reminiscence) context; this holds for observed as well as adolescent‐perceived parenting behaviors. Moreover, an emotional context elicits specifically more observable autonomy support. It is thus important to consider the context when evaluating parenting behaviors.

Second, adolescents with depression report more negative parenting behaviors than their HC peers, whereas there is no difference in observed parenting behaviors. Generally, the perception of the adolescent forms the reference point for therapy. It should be kept in mind that the adolescent's perception is not equal to the actual behavior of the parent. Along the lines of cognitive behavioral therapy, therapists could reflect with the adolescent on what biases might be set in motion for the adolescent in response to their parents' behavior. It may further be of value to reflect whether biases mainly originate from the depressive state and/or also from previous and daily life experiences of parenting behaviors and communication. Third, it is important to also consider observable parental psychological control when treating adolescents with depression. This parenting behavior worsens the affective state of adolescents with depression in interactions with their parent. Parents could be psycho‐educated and/or intervened on this behavior and the impact it has on their child.

### Strengths and limitations

To the best of our knowledge, the current study is the first to disentangle observed and adolescent‐perceived parenting behaviors concerning the same parent‐adolescent interactions, and thereby importantly adds to existing literature. It is further one of the first to link observations to adolescent‐perceived parenting behavior in daily life, indicating ecological validity of observing parenting behaviors in the lab. We were also able to include families with adolescents with a clinical depression, and a substantial number of fathers.

The current study has several limitations. First, the sample of adolescents with depression was relatively small, limiting the power of aim two of the study. Second, some important sample characteristics should be noted. Adolescents with certain comorbid disorders (i.e., intellectual disability, psychosis, eating disorders, substance use disorders, and/or autism spectrum disorders) were excluded and results cannot be simply generalized to all adolescents with a depression. Third, in both samples (clinical and HC), adolescents and parents had to be willing to participate together, which may have led to a selection bias. And fourth, the intercoder reliability of observed psychological control for the double‐coded videos was moderate. This might be due to coding with two groups (for practical reasons of coding a dataset of 628 videos with available students) with separate intervision meetings and/or due to low variability in observed PC, with subsequent larger impact of deviations in scoring.

## CONCLUSION

To conclude, results of this multi‐method study highlight three key messages. First, the newly developed CASPCA coding system was reliable and showed ecological validity, and can thus be well used to assess observed parental autonomy support and psychological control in parent‐adolescent interactions across different contexts in research. Second, adolescents with depression (vs. HC) perceived their parents somewhat more negative, but their parents did not behave differently (from HC) when interacting with the adolescent, indicating a negativity bias of adolescents with depression. And third, although parents of adolescents with depression did not express more observable psychological control, their expressed psychological control was followed by their child feeling worse, which was not the case for HC adolescents. Parents could be psycho‐educated (and intervened) on this behavior and its impact in a clinical setting.

## FUNDING INFORMATION

The study is funded by a personal grant awarded to Bernet Elzinga by the Netherlands Organization for Scientific Research (NWO‐VICI: Unraveling the Impact of Emotional Maltreatment on the Developing Brain, 453‐15‐006). The funding source was not involved in study design, analysis of data, interpretation of results, and writing of the report.

## CONFLICT OF INTEREST STATEMENT

The authors declare they have no competing or potential conflicts of interest.

## PATIENT CONSENT

Participants signed informed consent, and both parents with legal custody signed additional informed consent in case their child was younger than 16 years.

## Supporting information


Appendix S1

## Data Availability

Data available upon request from the corresponding author.
