# Supplementary material for: Balancing boundaries: Observed parental autonomy support and psychological control in the context of parent‐adolescent interactions and adolescent depression
Source: J Res Adolesc. 2025 Jan 26;35(1):e70003. doi: 10.1111/jora.70003 (PMC11771583; doi:10.1111/jora.70003)
Supplement: Supplementary file 1 — Appendix S1 [file JORA-35-0-s001.docx]

**Supplementary Information**

**Manuscript title:** Balancing boundaries: Observed parental autonomy support and psychological control in the context of parent-adolescent interactions and adolescent depression

**Journal:** Journal of Research on Adolescence

**Authors:** Wentholt, W. G. M., Meurs, E. H. A., Janssen, L. H. C., van Houtum, L. A. E. M., Wever, M. C. M., Tollenaar, M. S., Alink, L. R. A., & Elzinga, B. M.

**Correspondence:** Wilma G. M. Wentholt, Leiden University, Faculty of Social and Behavioral Sciences, 2333 AK Leiden, the Netherlands. E-mail: [w.g.m.wentholt@fsw.leidenuniv.nl](mailto:w.g.m.wentholt@fsw.leidenuniv.nl). ORCID ID: 0000-0001-9713-3384

**Supplementary Methods**

**Participants**

Data of 192 of the 210 parent-adolescent dyads (91.4%) were complete on all main variables. The reminiscence task was not assessed for one of the HC families (*n* = 2 parents) because the task was too stressful for the adolescent. Therefore, observed, perceived, and affect data were missing for this task. Data of adolescent-perceived parental listening/understanding and criticism/dominance was missing for five HC parents (n = 2 EPI, n = 3 REM) and one parent of an adolescent with depression (problem solving task), and data of adolescent positive and negative affect was missing for six HC adolescents (*n* = 1 baseline, *n* = 2 event planning task, *n* = 3 reminiscence task), because the researcher forgot to assess these measures in between the interaction tasks. Mean levels of adolescent-perceived listening/understanding and criticism/dominance in daily life were missing for six parents of HC adolescents, because there was an error in scaling of the items in the application (*n* = 2 from one family) or the adolescent had not filled out any of the questions on parental behavior (*n* = 4 fathers). We performed Little’s MCAR test (Little, 1988) to check whether data were missing completely at random, which was the case (χ²(132) = 88.59, *p* = .999).

**Measures**

***Depression Symptomatology***

The Patient Health Questionnaire-9 (PHQ-9) was used to assess depression symptomatology of adolescents and parents. The PHQ-9 is a well-validated and frequently used questionnaire, for which cut-off scores have been determined representing mild, moderate, moderately severe, and severe depression (Kroenke et al., 2001). A higher continuous score represents higher depression symptomatology.

Adolescents self-reported ten items on their depressive symptomatology in the past two weeks (e.g., “How often have you been bothered by feeling down, depressed, or hopeless?”) on a 4-point Likert scale (0 = not at all, 1 = some days, 2 = more than half of the days, 3 = almost every day). One item of the original PHQ-9 was split into two items to avoid ambiguity: “How often have you been bothered by moving and speaking slower than usual?” and “How often were you so fidgety or restless that you moved around more than usual?”. The item with the highest score was selected in further data processing. The final nine items were summed into the total score of depression symptomatology. Internal reliability in the sample with adolescents with depression was acceptable (Cronbach’s α = .76) and in the HC sample was questionable (Cronbach’s α = .62).

Parents self-reported ten items on their depressive symptomatology in the past two weeks (e.g., “How often have you been bothered by little interest or pleasure in doing things?”) on a 4-point Likert scale (0 = not at all, 1 = some days, 2 = more than half of the days, 3 = almost every day). The original nine items of the PHQ were used, with one additional item if the parent had experienced one or more items at some or more days (i.e., impact on daily functioning; 0 = not at all, 1 = somewhat, 2 = very, 3 = extremely). This final item was not included in the further data processing. The original nine items were summed into the total score of depression symptomatology. Internal reliability in the sample with adolescents with depression was good to excellent (mothers’ Cronbach’s α = .90; fathers’ Cronbach’s α = .89) and in the HC sample was acceptable to good (mothers’ Cronbach’s α = .77; fathers’ Cronbach’s α = .90).

***Parental Bonding***

The Parental Bonding Inventory was used to assess the parent-adolescent bonding. The PBI is a well-validated and frequently used questionnaire (e.g. Tam & Yeoh, 2008). The original PBI consists of two subscales (care, control), but a three-factor solutions has been shown to have better psychometric properties (Kullberg et al., 2020). Participants self-reported on 25 items on a 4-pointscale (0 = (totally) true, 3 = (totally) untrue). Adolescents reported on their mothers’ and fathers’ bonding separately, mothers and fathers separately reported on their own bonding towards their child. Data was missing for one father of the HC families, resulting in a total of 68 fathers of HC adolescents reporting on their own bonding. One of the items (of the Autonomy granting subscale) was translated incorrectly (“wants me to grow up” instead of the original “does not want me to grow up”) and excluded in current analyses. Items were recoded if necessary and summed into three subscales: Care (12 items; e.g. “My [mother/father] spoke to me in a warm and friendly voice”), Overprotection (6 items; e.g. “My [mother/father] felt I could not look after myself unless they were around”), and Autonomy granting (5 items; e.g. “My [mother/father] liked me to make my own decisions”). Note that the PBI subscale on autonomy concerns autonomy granting and not autonomy support, and thus differs from the construct of the coding system in the current study. A higher score represents higher levels of the respective bonding subscale (e.g., higher score on Care, represents *higher* levels of parental care).

Internal reliability (with Cronbach’s α) of Care in the sample with adolescents with depression was questionable to good (adolescents about mother α = .88, adolescents about father α = .86, mothers Cronbach’s α = .82, fathers Cronbach’s α = .66) and in the HC sample was questionable to good (adolescents about mother α = .83, adolescents about father α = .86, mothers Cronbach’s α = .74, fathers Cronbach’s α = .69). Internal reliability of Overprotection in the sample with adolescents with depression was unacceptable to acceptable (adolescents about mother α = .71, adolescents about father α = .42, mothers Cronbach’s α = .71, fathers Cronbach’s α = .49) and in the HC sample was unacceptable (adolescents about mother α = .52, adolescents about father α = .48, mothers Cronbach’s α = .59, fathers Cronbach’s α = .44). Internal reliability of Autonomy granting in the sample with adolescents with depression was questionable to excellent (adolescents about mother α = .91, adolescents about father α = .80, mothers α = .65, fathers α = .68) and in the HC sample was questionable to acceptable (adolescents about mother α = .72, adolescents about father α = .60, mothers α = .61, fathers α = .65).

***Adolescent Psychological Disorders***

The semi-structured diagnostic interview Kiddie-Schedule for Affective Disorders and Schizophrenia – Present and Lifetime (K-SADS-PL; Kaufman et al., 1996; Reichart et al., 2000) was used to diagnose adolescents’ psychological disorders. The K-SADS was assessed with adolescents with depression (at that point indicated) prior to the research day by trained psychologists of the recruiting mental health care facility (i.e., diagnostic information could then be included in treatment of the adolescent; *n* = 23) or by trained researchers (*n* = 12). The K-SADS was assessed with HC adolescents during the research day. The interview was used to check in- and exclusion criteria and current comorbid disorders.

***Indication of Parental Psychological Disorders***

The brief Mini International Neuropsychiatric Interview (MINI, version 5.0.0; Sheehan et al., 1998) was used to indicate present and lifetime parental psychological disorders. The MINI was assessed during the research day by trained researchers. This brief interview includes semi-structured questions on (symptoms of) a variety of psychological disorders, thereby indicating possible (i.e., not diagnosing) clinical levels. In context of the current study we categorized the data as indications of current and past depressive disorders (major depressive disorder or dysthymia) and other psychological disorders.

**Coding Manual – Coding Parental Autonomy Support and Psychological Control in Adolescence (CASPCA)**

**Concepts and Definitions**

The current coding system was designed to quantify parental autonomy-supportive and psychologically controlling behaviors directed at their child (i.e., adolescents) as observed in interaction tasks. We will start this coding manual with the definitions and some theoretical background of the central concepts.

The development of autonomous functioning is considered as a crucial developmental process for adolescents (Soenens et al., 2007). Autonomy is one of the three basic psychological needs as stated in the self-determination theory (Ryan & Deci, 2000). The concept can be defined as “a sense of self-reliance, a belief one has control over his/her life, and subjective feelings of being able to make decisions without excessive social validation” (Sessa & Steinberg, 1991, p. 42). Autonomy supportive relationships are thought to encourage such self-determined functioning in adolescents and are critical for adolescents’ well-being (Grolnick et al., 1991).

A parent who functions highly on autonomy-supportive behavior is both structuring and supportive and can clearly explain why certain decisions are being made by the parent, while at the same time allowing the adolescent to express his/her own opinion and feelings (Soenens & Vansteenkiste, 2010). Parental autonomy-support refers to the degree to which the parent empathizes with the child’s perspective, to which there is an openness in the communication about potentially different perspectives of the parent and adolescent, and to which the parent encourages their child to take initiative and offer meaningful explanation when choice is limited (Soenens & Vansteenkiste, 2010).

Psychological control has been defined in terms of manipulation and intrusion into children’s feelings and thinking through behaviors such as invalidating children’s feelings and pressuring them to think in particular ways (Barber et al., 2005; Lansford et al., 2014). Psychological control can be considered a restriction of autonomy or an intrusion by parents into adolescents’ establishment of autonomy (Lansford et al., 2014). Coming from the self-determination theory, parental control refers to attempts at forcing children to meet demands, solving problems for children and taking a parental rather than child’s perspective (Deci & Ryan, 2000). Psychologically controlling parents regulate their child’s activities, are overprotective, invalidate their child’s emotions, induce guilt, and/or instruct their child how to feel, think, and behave (Donatelli et al., 2007; Elzy, 2013; Rogers et al., 2003).

Even though the concepts of parental autonomy-supportive and psychological controlling behaviors are related, several studies indicate that these concepts must be seen as separate constructs rather than a continuum (Barber et al., 2005; Hauser Kunz & Grych, 2013). Although parents who frequently engage in psychological control are unlikely to actively promote their child’s autonomy (and visa), the absence of psychological control does not imply the presence of autonomy-support. Likewise, parents who are low in autonomy-support do not by definition engage in high levels of psychological control. Rather, parents may discourage autonomous thinking and feeling explicitly by setting and reinforcing established standards for their child’s behavior (i.e., behavioral control) instead of using manipulative and coercive methods (Hauser Kunz & Grych, 2013).

**Interaction Tasks and Coding Procedure**

With the current coding system we aim to quantify parental autonomy-support and psychological control behaviors separately in videotaped dyadic parent-adolescent interactions. A higher score represents higher levels of the particular behavior. Thus a higher score on the autonomy-support scale indicates higher levels of positive, desirable behavior, and a higher score on the psychological control scale indicates higher levels of negative, undesirable behavior. Subsequently, the construction of these two scales differs.

The coding system is applicable to three interaction tasks. Parent and adolescent are placed at a table in a 90° angle, with one camera pointed at the parent and one at the adolescent.

- Problem solving task (10 minutes): The dyad is instructed to discuss and solve a topic of discussion they have encountered in the four weeks prior to the lab visit. Parent and adolescent independently report the frequency and intensity of several possible topics of discussion via the Issues Checklist at the start of the laboratory visit. The researcher selects the three topics that were reported to be the most frequent and intense topics of discussion.
- Event planning task (6 minutes): The dyad is instructed to plan a trip (i.e., weekend) together that they would both enjoy and with an unlimited budget.
- Reminiscence task (6 minutes): The adolescent is asked to share an emotional event (reported at the start of the laboratory visit) with their parent. Preferably this concerns an event the parent has no knowledge of, and that the parent was not part of.

Adolescents participate with either one or two parents, depending on whether the second parent (1) is involved in the daily life/upbringing of the adolescent and (2) is willing to participate. If both parents participate, the adolescent will complete the interaction tasks twice; once with each parent. Thus in total there are three videotaped interactions of families in which only one parent participates, and six videotaped interactions for families with two participating parents. All families end the interaction tasks with a Jenga task (working together to build an as high as possible Jenga tower; 10 minutes) in a triad, but the current system is not applicable to this task.

The following general coding rules should be followed:

- The autonomy-support and psychological control scales each consist of three subscales. Each subscale gets assigned one score per interaction task, next the mean is computed for the final score of autonomy-support and psychological control separately.
- The scale ‘Explaining motivations’ is only scored for the problem solving task, and is not applicable (NA) to the event planning or reminiscence task.
- Coding is done on a 9-point scale. The scores 1, 3, 5, 7, and 9 are described in detail below, and the scores 2, 4, 6, and 8 can be used if the behavior of the parent seems to fall in between two scores (e.g., when one element is nearly optimal, making score 5 too low, and the other element is clearly non-optimal, making score 7 too high, score 6 can be assigned).
- The coder first watches each video with regular pauses (e.g., every minute) and takes notes. A score can be assigned based on these notes. The coder should then watch the video again, without pausing it, to check whether the score indeed represents the behaviors. With this approach we aim to reduce a bias based on general feeling/likeability that may be evoked when watching the full video.
- One coder is never allowed to code the parental behavior of multiple interaction tasks, as this may lead to a bias when coding the following tasks.
- Intervision meetings will be held to discuss doubts and promote intercoder reliability.

**Coding Parental Autonomy Support**

***I. Encouraging Input of Adolescent***

This category is intended to capture the extent to which the parent encourages the input of the adolescent throughout the interaction, as expressed in two elements. First, the parent (non)verbally encourages the adolescent to give input. Second, the parent demonstrates patience.

- (Non)verbal encouragement is demonstrated by stimulating the adolescent to give input during the interaction, for example, by asking open-ended questions, providing opportunities for choices (e.g., letting adolescent decide who starts the interaction), by creating transitions if the adolescent is struggling to find input (e.g., introducing the topic or asking open or closed questions to help the adolescent getting started), or by non-verbal behavior such as facing the adolescent and nodding.
- Patience is demonstrated by not interrupting the adolescent(‘s thought process) and actively listening (e.g., making eye-contact and having an active posture) to the adolescent or waiting for the adolescent to give input.

9. The parent consistently encourages the adolescent’s input, as observed in both elements.

- (Non)verbal encouragement is optimal.
- AND Demonstrating patience is optimal.

**7.** The parent in general encourages the adolescent’s input, but only *one* of the elements is less optimal.

- (Non)verbal encouragement is less optimal if the parent misses some (subtle) opportunities to (non)verbally encourage the adolescent or is not able to create transitions if the adolescent struggles to find input. In those cases the parent does try to do so but the attempt does not fully fit the adolescent’s mood, attention, or understanding.
- OR Demonstrating patience is less optimal if the parent, for example, in some cases interrupts the adolescent or does not wait for the adolescent to start giving input.

5. The parent encourages the adolescent’s input to some extent. However, both elements are less optimal but not at the other end of the continuum.

- (Non)verbal encouragement is less optimal if the parent misses some (subtle) opportunities to (non)verbally encourage the adolescent or is not able to create transitions if the adolescent struggles to find input. In those cases the parent does try to do so but the attempt does not fully fit the adolescent’s mood, attention, or understanding.
- AND Demonstrating patience is less optimal if the parent, for example, in some cases interrupts the adolescent or does not wait for the adolescent to start giving input.

3. The parent only marginally encourages the adolescent’s input. The parent demonstrates the elements a few times but more often does not show these behaviors. Or one of the elements is fully absent while the other is observed to some extent.

- (Non)verbal encouragement is absent more often than it is demonstrated, if the parent (non)verbally encourages the adolescent only in some occasions.
- AND Patience is absent more often than it is demonstrated. For example, the parent may demonstrate patience after the adolescent asks for space to give input. But in general the parent interrupts the adolescent and/or is speaking a lot during the conversation.
- OR One of the elements is fully absent, while the other is observed to some extent (i.e., from marginally to optimally). For example, a parent does give quite a few verbal encouragements but never demonstrates patience, meaning the adolescent is encouraged to think about his / her own perspective, but does not get any space to express it.

1. The parent (virtually) never encourages the adolescent to give input. The parent is consistently not demonstrating both elements.

- (Non)verbal encouragement is absent if the parent does not demonstrate any encouraging behavior such as asking open-ended questions, exploring the adolescent’s perspective, creating transitions, or providing a choice.
- AND Demonstrating patience is absent if the parent is either constantly interrupting the adolescent and is dominating the conversation, or not actively listening (e.g., no eye-contact and a passive posture).

***II. Explaining motivations behind parental advice, limits, and perspectives***

This category is intended to capture the extent to which the parent explains motivations or reasons for the advice, limits, and perspectives throughout the interaction, as expressed in two elements. First, the parent explains the motivations in a clear, calm, and respectful manner. Second, the parent takes notion of the mood, attention, and understanding of the adolescent while explaining the motivations.

- A clear, calm, and respectful manner is demonstrated by, for example, speaking with a calm tone of voice, and awareness that the motivations concern a personal perspective rather than the absolute truth.
- Taking notion of the mood, attention, and understanding of the adolescent is demonstrated by, for example, using appropriate language, asking whether the adolescent understands the parents’ motivations, and changing the strategy if the adolescent is not engaged in the conversation or does not understand the content.

*Note.* This category is only scored when coding the problem solving task and never when coding the event planning and reminiscence task (use NA on coding form). This category is not applicable due to the nature of these tasks: The parent ideally focuses on and explores the input and emotions of the adolescent, rather than focusing on his/her own ideas. If the parent explains his/her own motivations during these tasks, this can even impair the support for autonomy.

9. The parent consistently explains motivations behind advice, limits, and perspectives suited to the situation as observed in both elements.

- Optimal demonstration of a clear, calm, and respectful manner to explain motivations.
- AND Taking notion of the mood, attention, and understanding of the adolescent is optimal.

**7.** The parent explains motivations behind advice, limits, and perspectives as observed in both elements, but only *one* (not both) of the elements is less optimal.

- The clear, calm, and respectful manner to explain motivations is less optimal if the parent, for example, is overdoing the explaining by repeating argumentations or elaborating too much, or is somewhat emotional in his/her argumentation. However, the parent adjusts this behavior. The adjustment is either in response to the (non)verbal reaction of the adolescent or the parent is not persistent in the unclear and/or not calm (e.g., repetitive or elaborate) manner.
- OR Taking notion of the mood, attention, and understanding of the adolescent is less optimal if the adolescent is not receptive (i.e., due to mood, attention, and/or understanding) to the explanation, and the parent does not adapt to this promptly. The parent, for example, explains his/her motivations while the adolescent expresses a negative mood (e.g., sighing or rolling with eyes). The parent focuses on his/her own motivations prior to attending to the adolescents’ affective expressions, and may need some time to note and respond to the adolescents’ unreceptiveness. However, the element is less optimal and not absent, thus although the parent may need some time, (s)he is able to adjust his/her behavior and explanations to the adolescent.

5. The parent explains the motivations behind advice, limits, and perspectives to some extent. However, both elements are less optimal but not at the other end of the continuum.

- The clear, calm, and respectful manner to explain motivations is less optimal if the parent, for example, is overdoing the explaining by repeating argumentations or elaborating too much, or is somewhat emotional in his/her argumentation. However, the parent adjusts this behavior. The adjustment is either in response to the (non)verbal reaction of the adolescent or the parent is not persistent in the unclear and/or not calm (e.g., repetitive or elaborate) manner.
- AND Taking notion of the mood, attention, and understanding of the adolescent is less optimal if the adolescent is not receptive (i.e., due to mood, attention, and/or understanding) to the explanation, and the parent does not adapt to this promptly. The parent, for example, explains his/her motivations while the adolescent expresses a negative mood (e.g., sighing or rolling with eyes). The parent focuses on his/her own motivations prior to attending to the adolescent’s affective expressions, and may need some time to note and respond to the adolescent’s unreceptiveness. However, the element is less optimal and not absent, thus although the parent may need some time, (s)he is able to adjust his/her behavior and explanations to the adolescent.

3. The parent only marginally explains the motivations behind advice, limits, and perspectives. The parent demonstrates the elements a few times but more often does not show these elements, or barely explains any motivations behind advice, limits, and perspectives. Or one element is fully absent and the other is observed to some extent.

- The clear, calm, and respectful manner is absent more often than it is demonstrated. In some occasions the parent may explain his/her perspective in a clear, calm, and respectful manner, but more often the parent does not explain his/her perspective at all or (for example) does so in an emotional manner or presenting these explanations as the absolute truth.
- AND The parent takes no notion of the mood, attention, and understanding of the adolescent more often than he/she does take notion of this. In some occasions the parent may be attuned to the adolescent but more often the parent explains his/her motivations (for example) too elaborate or repetitive, resulting in a more plea-like or whining argumentation.
- OR One of the elements is fully absent, while the other is observed to some extent (i.e., from marginally to optimally). For example, a parent explains his/her motivations in a very clear, calm, and respectful manner, but the adolescent is not receptive to the parent’s reasoning at all (e.g., rolling with eyes, sighing, showing other non-verbal disinterest) and the parent does not adapt the motivations at all.

1. The parent (virtually) never explains motivations behind advice, limits, and perspectives at all. The parent is consistently not demonstrating the elements.

- The clear, calm, and respectful manner is absent if the parent either never explains motivations behind advice, limits, and perspectives, or if the manner of explaining consistently is, for example, emotional or presented as being the absolute truth.
- AND Taking notion of the mood, attention, and understanding of the adolescent is absent if the parent either never explains the motivations or consistently does this, for example, in too elaborate or repetitive reasoning, resulting in a more plea-like or whining argumentation.

***III. Receptive to expressions made by adolescent***

This category is intended to capture the extent to which the parent is receptive (both verbal and nonverbal) to the adolescent’s expressions (either verbal or nonverbal, affective or cognitive) during the interaction, as expressed in two elements. First, the parent actively accepts the adolescent’s expressions. And second, the parent tries to relate to and understand the adolescent’s expressions.

- Active acceptance is demonstrated if the parent notices the adolescent’s expressions, is tolerant, and engages in the interaction in a positive, non-defensive manner. In case of affective expressions of the adolescent, the parent empathically responds to emotions and sensitive topics and is nonverbally accepting (e.g., tone of voice, facial expression), while in case of cognitive expressions by the adolescent, the parent accepts that the adolescent has ideas and perspectives of his/her own.
- Trying to relate to and understand the adolescent’s expressions is demonstrated if the parent is reflecting on (e.g., by reformulating the adolescent’s expressions) and responding to the expressions (e.g., by asking in-depth questions or adding own interpretations). Furthermore, the parent allows to adolescent to express at his/her own pace, without (for example) immediately asking new questions. Concerning affective expressions, the parent relates to the emotions, rather than the situation or the topic (e.g., giving advice or asking details of more practical issues of the situation). In case of negative or ambiguous expressions the parent attends to these in an explicit manner, but in case of positive expressions (mainly during the event planning task), this may also be implicit, for example by sharing in enthusiasm.

9. The parent is consistently receptive to expressions made by the adolescent and adequately responds to them, as observed in both elements.

- The active acceptance is optimal.
- AND The attempt to relate to and understand the adolescent’s expressions is optimal.

7. The parent in general is receptive to expressions made by the adolescent and adequately responds to them, but only *one* (not both) of the elements is less optimal.

- The active acceptance is less optimal if the parent is less tolerant to part of the adolescent’s expressions or if the acceptance in general is less accepting. For example, the parent is not able to engage in the conversation in a fully positive and non-defensive manner. These aspects are less optimal and not absent, and do not concern a negative manner.
- OR The attempt to relate to and understand the adolescent’s expressions is less optimal if the parent is less reflecting or responding. For example, the parent responds superficially rather than in-depth in some cases. Concerning negative or ambiguous emotions this can also mean the parent tries to relate to and understand them in an implicit manner, by sharing or supporting the emotions rather than further exploring them. It is important that this implicit attendance is in line with the emotions or needs of the adolescent; the adolescent is responsive and shows signs of comfort.

5. The parent is receptive to expressions made by the adolescent and adequately responds to them to some extent, and never misses the more obvious opportunities. However, both elements are less optimal but not at the other end of the continuum.

- The active acceptance is less optimal if the parent is less tolerant to part of the adolescent’s expressions or if the acceptance in general is less accepting. For example, if the parent is not able to engage in the interaction in a fully positive and non-defensive manner. These aspects are less optimal and not absent and do not concern a negative manner.
- AND The attempt to relate to and understand the adolescent’s expressions is less optimal if the parent is less reflecting or responding. For example, the parent responds superficially rather than in-depth in some cases. Concerning negative or ambiguous emotions this can also mean the parent tries to relate to and understand them in an implicit manner, by sharing or supporting the emotions rather than further exploring them. It is important that this implicit attendance is in line with the emotions or needs of the adolescent; the adolescent is responsive and shows signs of comfort.

3. The parent is only marginally receptive to expressions made by the adolescent. The parent demonstrates the elements a few times but more often does not show these elements. Or one element is absent and the other is observed to some extent.

- In some occasions the parent may actively accept the adolescent’s expressions, but more often the parent is not reacting to or even rejecting, verbally or nonverbally (e.g., gruff or cynical tone of voice or facial expression) the adolescent’s expressions.
- AND The attempt to relate to and understand the adolescent’s expressions is more often absent than demonstrated by the parent. In some occasions the parent may, explicitly or implicitly, relate to and understand the adolescent’s expressions, but more often the parent (for example) does not further explore the expressions. Or, in case of emotional behavior or content, focuses on practical aspects of the situation or advices the adolescent.
- OR One of the elements is fully absent, while the other is observed to some extent (i.e., from marginally to optimally). For example, a parent who actively accepts all or most of the adolescent’s expression, but never attempts to relate to or understand them.

1. The parent is (virtually) never receptive to expressions made by the adolescent. The parent is consistently not demonstrating the elements.

- The active acceptance is absent if the parent does not notice the expressions (i.e., as far as this can be observed by the coder) or does not attend to them, but rather, for example, ignores them, ‘correcting’ the adolescent a lot, or elaborates on his/her own perspectives.
- AND The attempt to relate to and understand the expressions is absent if the parent does not try to further explore adolescent’s expressions, but is rather, for example, consistently interrupting the adolescent, very impatient if the adolescent tries to express his/her affections or cognitions, or is changing the topic.

**Coding Parental Psychological Control**

***I. Constraining verbal expressions***

This category is intended to capture the extent to which the parent is constraining verbal expressions of the adolescent throughout the interaction, as expressed in three elements. First, the parent demonstrates dominating behaviors. Second, the content of the parents’ expressions is dominant. And third, the parent demonstrates disinterest.

- Dominant behavior is demonstrated if the parent shows behaviors that interfere with the adolescent or otherwise restrict the adolescent to express. Examples are asking leading questions, interrupting the adolescent, speaking a lot during the interaction an thereby restricting the adolescent to express, answering their own questions, speaking for the other, or switching topics.
- Dominant content is demonstrated if the verbal expressions of the parent have a leading content, leaving no space for expressions or reasoning of the adolescent. Examples of such dominating content are ‘should/ought statements’, lecturing, advising, and suggesting solutions.
- Disinterest means the parent is ignoring the adolescent’s comment or non-verbally communicating disinterest (e.g., facing away from the adolescent aiming to ignore the adolescent).

Low intensity episodes:

- Dominating behavior. The parent has a somewhat dominant attitude and tone of voice.
- Dominating behavior. While speaking, the parent does not allow the adolescent to interrupt him/her, because he/she wants to elaborate on his/her own point. The parent had already allowed the adolescent to express his/her thoughts or feelings, but next is speaking for quite some time and then does not allow the adolescent to interrupt/take over.
- Dominating content. The parent is overdoing the explanation of motivations, resulting in a mild form of lecturing.
- Dominating content. The parent keeps track on his/her own reasoning or suggestions. The parent listens to the adolescent but next elaborates on his/her own reasoning.
- Dominating content. The parent is somewhat leading in the content of the interaction, but the parent is able to adjust this to the adolescents’ input relatively quickly.
- Dominating content. The parent suggests solutions to the discussed situation (advising/wanting to solve a situation for the adolescent), after first exploring the adolescent’s perception. The solutions are suggestive rather than compelling, but the adolescent did not ask the parent to think about a possible solution nor is (s)he receptive to the suggested solution.
- Disinterest. The parent initially ignores the adolescent’s suggestions, but reacts to these suggestions at a later moment out of own motivation.

Mild intensity episodes:

- Dominating behavior. The parent abruptly interrupts the adolescent or switches topic, in a non-pressuring and brief manner.
- Dominating behavior. While speaking, the parent does not allow the adolescent to interrupt him/her, because he/she wants to elaborate on his/her own point. This behavior is scored as a mild intensity episode if the parent is rather leading in the conversation. Meaning the parent has already been speaking for quite some time or the adolescent has not yet had a chance to speak up to the moment the adolescent tries to interrupt the parent. However, after a reasonable while (e.g., finishing the sentence), the parent does encourage or allow the adolescent to give input.
- Dominating content. The parent does listen to the ideas of the adolescent (e.g., where to go during event planning interaction), adds own ideas, and chooses one of the proposed ideas themselves rather than letting the adolescent or mutually decide.
- Dominating content. The parent suggests solutions to the discussed situation (advising/wanting to solve a situation for the adolescent), without first exploring the adolescents’ perception. The solutions are suggestive rather than compelling, and the adolescent did not ask the parent to think about a possible solution nor is (s)he receptive to the suggested solution.
- Dominating content. Keeping track on one’s own reasoning. The parent listens to the adolescent but next elaborates on their own reasoning. Furthermore, the parent is advising, lecturing, or setting boundaries in this elaboration.
- Disinterest. The parent initially ignores the adolescent’s suggestions, but reacts to these suggestions if the adolescent keeps repeating them.

Moderate intensity episodes:

- Dominating behavior. The parent abruptly interrupts the adolescent or switches the topic (e.g., by shushing the adolescent). This behavior occurs in a pressuring and leading manner. However, the parent does not cross an emotional/vulnerable moment of the adolescent or a sensitive topic.
- Dominating behavior. While speaking, the parent does not allow the adolescent to interrupt him/her, because he/she want to elaborate on his/her own point. The parent is leading in the interaction: the adolescent has not yet had a chance to express his/her thoughts or feelings, and the parent has been speaking for quite some time. Furthermore, the parent does not stop their own reasoning within a reasonable timeframe to let the adolescent express his/her thoughts or feelings.
- Dominating content. The parent suggests solutions to the discussed situations (advising/wanting to solve a situation for the adolescent), without first exploring the adolescent’s perception. Furthermore, the solutions are presented compelling rather than suggestive, and the adolescent did not ask the parent to think about a possible solution nor is (s)he receptive to the suggested solution.
- Disinterest. The parent ignores suggestions of the adolescent, neither reacts to them (out of own motivation or because of repetition adolescent) at a later moment.

Strong intensity episodes:

- Dominating behavior. The parent abruptly interrupts the adolescent. This behavior occurs in a pressuring and leading manner, and the parent crosses an emotional/vulnerable moment of the adolescent or a sensitive topic.
- Dominating content. The parent suggests solutions to the discussed situations (advising/wanting to solve a situation for the adolescent), without first exploring the adolescents’ perception, and the adolescent did not ask the parent to think about a possible solution nor is (s)he receptive to the suggested solution.. Furthermore, the solutions are presented compelling rather than suggestive and the parent keeps pressuring on these solutions (high and persistent level of leading content).

9. The parent:

- Consistently constraint the adolescent’s verbal expressions, many parental behaviors contain some type of constraining (low, mild, and/or moderate intensity).
- AND Demonstrates one or several strong intensity episodes occur.

7. The parent:

- Frequently demonstrates low, mild, and/or moderate intensity constraining episodes
- AND/OR Demonstrates one or more strong intensity constraining episodes.

5. The parent:

- Demonstrates infrequent mild intensity episodes.
- AND/OR Demonstrates one brief moderate intensity episode.

3. The parent demonstrates infrequent low intensity episodes.

1. The parent never constrains the adolescents’ verbal expressions.

***II. Guilt induction***

This category is intended to capture the extent to which the parent is demonstrating guilt inducing behaviors (i.e., attempts to force behaviors and thoughts) throughout the interaction, as expressed in two elements. First, the parent is making the adolescent unreasonably responsible. And second, the parent is prioritizing his/her own perspectives and needs.

- Making the adolescent unreasonably responsible is demonstrated if the parent is making the adolescent responsible for a situation that is hard to resolve. Pointing out the responsibility of the adolescent is making the adolescent responsible but not necessarily negative, guilt inducing behavior. However, it *is* coded if the parent is making the adolescent responsible for a situation the adolescent is not actually responsible for, or cannot resolve. This concerns behaviors that are within the relationship between parent and adolescent (e.g., conflict between parents about how to raise the adolescent) and *not* if it concerns a reasonable responsibility (e.g., making the adolescent responsible for a mess (s)he did not actually make).
- Prioritizing own perspectives and needs is demonstrated if the parent is placing his/her own perspectives and needs above those of the adolescent; the adolescent’s behavior/feelings are bothering the parent. This concerns behaviors that are within the relationship between parent and adolescent and *not* if it concerns more practical aspects (e.g., choosing where to go without exploring the adolescent’s perspective during the Event planning interaction). For example, by pointing out the parent has suffered a lot for the adolescent, mirroring the adolescent’s problems to those of the parent (i.e., pointing out own problems are worse than those of the adolescent), or not allowing the adolescent to express emotions because the parent is emotional him/herself.

Low intensity episodes:

- Making the adolescent unreasonably responsible. The parent describes a situation resulting in a responsibility placed by the adolescent; this parental behavior occurs in a brief and calm (i.e., no emotional tone of voice or attitude) manner.
- Prioritizing own perspectives and needs. The parent describes a situation or behaves in a way that places his/her own perspectives and needs above those of the adolescent; this parental behavior occurs in a brief and calm (i.e., no emotional tone of voice or attitude) manner.

Mild intensity episodes:

- Making the adolescent unreasonably responsible. The parent describes a situation resulting in a responsibility placed by the adolescent; this parental behavior occurs in a brief and somewhat emotional (i.e., tone of voice or attitude) manner.
- Prioritizing own perspectives and needs. The parent describes a situation or behaves in a way that places his/her own perspectives and needs above those of the adolescent; this parental behavior occurs in a brief and somewhat emotional (i.e., tone of voice or attitude) manner.

Moderate intensity episodes:

- Making the adolescent unreasonably responsible. The parent describes a situation resulting in a responsibility placed by the adolescent; this parental behavior occurs in a somewhat pressuring and clearly emotional (i.e., tone of voice or attitude) manner.
- Prioritizing own perspectives and needs. The parent describes a situation or behaves in a way that places his/her own perspectives and needs above those of the adolescent; this parental behavior occurs in a somewhat pressuring and clearly emotional (i.e., tone of voice or attitude) manner.

Strong intensity episodes:

- Making the adolescent unreasonably responsible. The parent describes a situation resulting in a responsibility placed by the adolescent; this parental behavior occurs in a clearly pressuring and emotional (i.e., tone of voice or attitude) manner.
- Prioritizing own perspectives and needs. The parent describes a situation or behaves in a way that places his/her own perspectives and needs above those of the adolescent; this parental behavior occurs in a clearly pressuring and emotional (i.e., tone of voice or attitude) manner.

9. The parent:

- Consistently demonstrates guilt inducing behaviors, many parental behaviors contain some type of guilt induction (low, mild, and/or moderate intensity).
- AND Demonstrates one or several strong intensity episodes occurs.

7. The parent:

- Frequently demonstrating low, mild, and/or moderate intensity guilt inducing episodes.
- AND/OR Demonstrates one or more strong intensity guilt inducing episodes.

5. The parent:

- Demonstrates infrequent mild intensity episodes.
- AND/OR Demonstrates one brief moderate intensity episodes.

3. The parent demonstrates infrequent low intensity episodes.

1. The parent never demonstrates guilt inducing behaviors.

***III. Invalidating emotions***

This category is intended to capture the extent to which the parent is demonstrating behaviors that invalidate the adolescent’s emotions, as expressed in three elements. First, the parent demonstrates nonverbal signs that invalidate the adolescents’ emotions. Second, the parent assigns values to adolescent’s emotions. And third, the parent is minimalizing the adolescent’s emotions.

- Assigning values to emotions is demonstrated if the parent is filling in emotions for the adolescent (e.g., via mind-reading behaviors) or is stating that emotions have a certain value (e.g., wrong, incorrect, not normal, unimportant, weak).
- Minimalizing is demonstrated if the parent shows behavior (verbally or nonverbally) that (aims to) diminishes the adolescent’s emotions (e.g., stating the other is overreacting, stating the adolescent should forget about it and move on, minimalized enthusiasm of the adolescent by being flat/discouraging, ignoring the content, rolling eyes, tone of voice, sighing, or expressing positive feelings about a situation the adolescent has expressed to experience as negative).

Low intensity episodes:

- Minimalizing and nonverbal signs. The parent has a very flat, discouraging attitude or tone of voice at a moment of enthusiasm of the adolescent.
- Minimalizing. The parent ignores specific content of the adolescent. For example, by discussing practical aspects after an emotional expression of the adolescent.

Mild intensity episodes:

- Assigning values to emotions. The parent is filling in the emotions of the adolescent (i.e., mind reading) in a suggestive, non-pressuring, and brief manner. The parent does not check whether this fits the adolescent’s actual emotions. If the parent *does* check the adolescent’s perception/actual experience/emotions, the behavior is not coded here, because the behavior then promotes the exploration of the adolescents’ emotions.

Moderate intensity episodes:

- Assigning values to emotions. The parent is filling in the emotions of the adolescent (i.e., mind reading) in a certain context or situation, in a rather firm/direct, but non-pressuring and brief manner. The parent does not check whether this fits the adolescent’s actual emotions.
- Minimalizing. The parent states he/she has positive feelings about a situation or moment after the adolescent has said he/she has negative feelings about that situation or moment.

Strong intensity episodes:

- Assigning values to emotions. The parent is filling in the emotions of the adolescent (i.e., mind reading) in a certain context or situation, in a rather firm/direct, pressuring, and leading manner. The parent does not check whether this fits the adolescents’ actual emotions.

9. The parent:

- Consistently invalidates the adolescent’s emotions. Many parental behaviors contain some type of invalidating emotions (low, mild, and/or moderate intensity).
- AND Demonstrates one or several strong intensity episodes occur.

7. The parent:

- Frequently demonstrates low, mild, and/or moderate intensity behaviors that invalidate the adolescent’s emotions.
- AND/OR Demonstrates one or more strong intensity episodes of invalidating emotions.

5. The parent:

- Demonstrates infrequent mild intensity episodes.
- AND/OR Demonstrates one brief moderate intensity episodes.

3. The parent demonstrates infrequent low intensity episodes.

1. The parent never demonstrates behaviors that invalidate the adolescent’s emotions.

**PPN #**

**Coding Form**

| **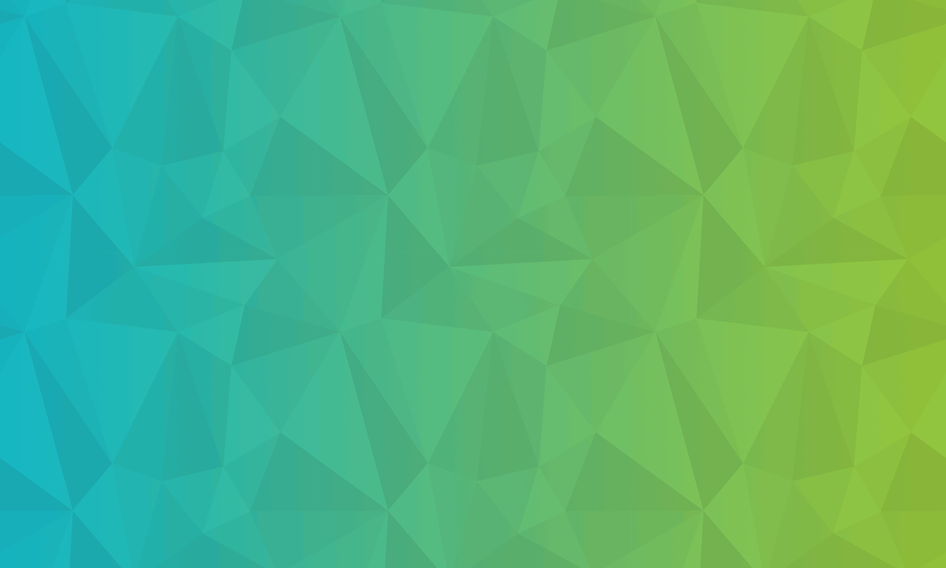Task** | PSI/EPI/REM | | | **Coder** |  |
| --- | --- | --- | --- | --- | --- |
| **Task as instructed?** | Yes / Partly / Not at all | | | **Date** |  |
| **Autonomy-supportive behaviors** | | **Score** | **Notes** | | |
| I. Encouraging input | |  |  | | |
| II. Explaining motivations | |  |  | | |
| III. Receptive to expressions | |  |  | | |
| **Psychologically controlling behaviors** | | **Score** | **Notes** | | |
| I. Constraining verbal expressions | |  |  | | |
| II. Guilt induction | |  |  | | |
| III. Invalidating feelings | |  |  | | |

| **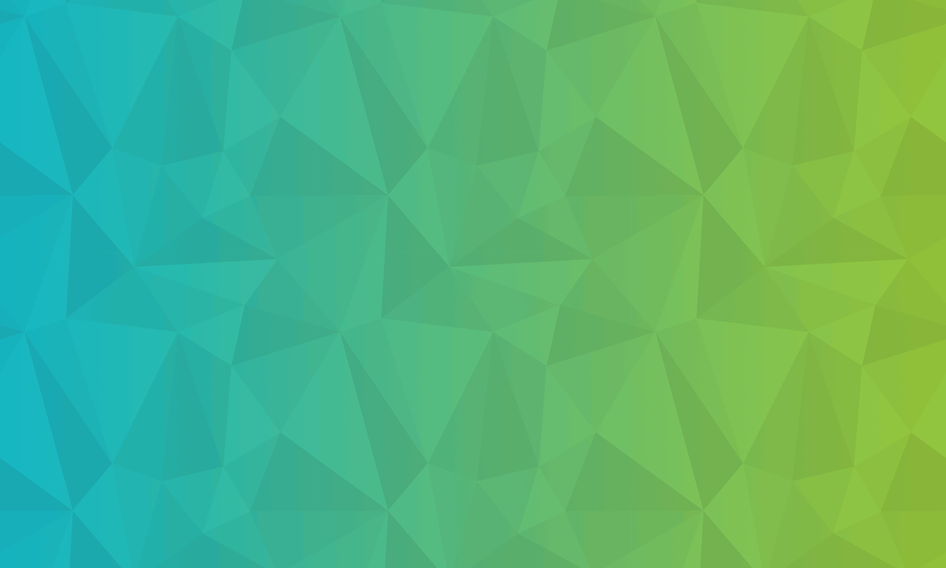Element** | **Notes** |
| --- | --- |
| **Autonomy-Support** | |
| (Non) verbal encouragement |  |
| Patience |  |
| Clear, calm, respectful |  |
| Adjusted to adolescent |  |
| Active acceptance |  |
| Relating, understanding |  |
| **Psychological Control** | |
| Dominating behavior |  |
| Dominating content |  |
| Disinterest |  |
| Unreasonable responsibility |  |
| Prioritizing own perspective |  |
| Assigning values |  |
| Minimalizing |  |

| **Time** | **Relevant behaviors** |
| --- | --- |
| Start fragment | **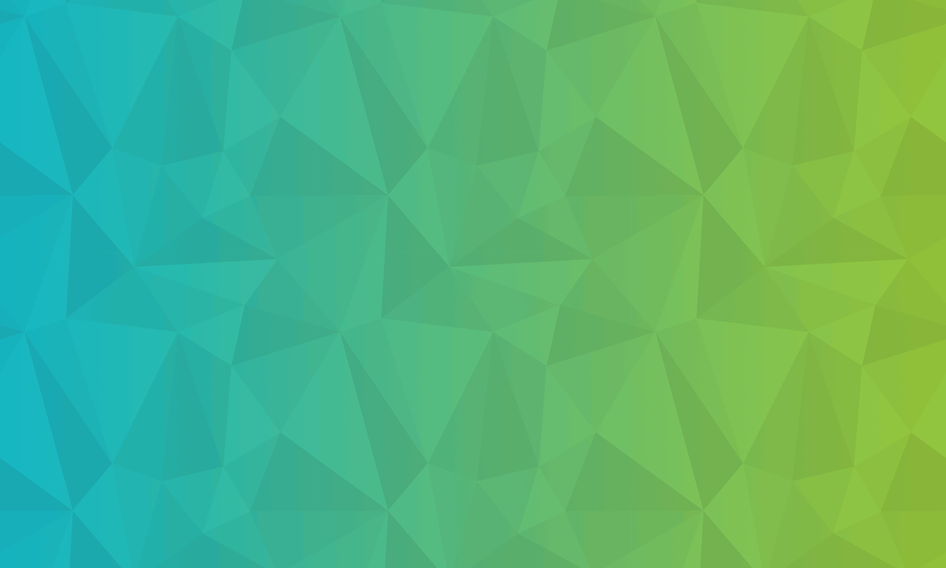** |
|  |  |
|  |  |
|  |  |
|  |  |
|  |  |
|  |  |
|  |  |
|  |  |
|  |  |
|  |  |
|  |  |
|  |  |
|  |  |
|  |  |
|  |  |
|  |  |
|  |  |
|  |  |
|  |  |
|  |  |
|  |  |
|  |  |
|  |  |

| **Supplementary Table 1**  *Subscales Coding System and Related Behaviors from Previous Studies* | |
| --- | --- |
| **Current coding system** | **Based on** |
| Parental Autonomy Support | |
| Encouraging input | Queries of other which are truly information seeking (Allen et al., 1994) |
|  | Engaged interaction (Allen et al., 1994) |
|  | Encourages child to express views (Hauser Kunz & Grych, 2013) |
|  | Encourages independent thinking (Hauser Kunz & Grych, 2013) |
|  | Provision of choice (Mageau et al., 2016; Wuyts et al., 2018) |
|  | Asking experience questions (Wuyts et al., 2018) |
|  | Authentic interest (Wuyts et al., 2018) |
|  | Awaiting [disclosure] (Wuyts et al., 2018) |
| Explaining motivations | Tolerates differences and disagreements (Hauser Kunz & Grych, 2013) |
|  | Provision of rationale for rules and demands (Mageau et al., 2016) |
| Receptiveness to input | Queries of other which are truly information seeking (Allen et al., 1994) |
|  | Receptive to statements made by child (Allen et al., 1994; Hauser Kunz & Grych, 2013) |
|  | Tolerates differences and disagreements (Hauser Kunz & Grych, 2013) |
|  | Acknowledgement of child’s feelings (Mageau et al., 2016) |
|  | Reflective listening (Wuyts et al., 2018) |
|  | Empathic understanding (Wuyts et al., 2018) |
| Parental Psychological Control | |
| Constraining expressions | Pressures other to agree (Allen et al., 1994; Mageau et al., 2016) |
|  | Distracting/ignoring/interrupting (Allen et al., 1994; Wuyts et al., 2018) |
|  | Constrain verbal expressions (Barber, 1996) |
|  | Closed questioning (Wuyts et al., 2018) |
|  | Using controlling language (Wuyts et al., 2018) |
|  | Commanding (Wuyts et al., 2018) |
|  | Unsolicited advising or lecturing (Wuyts et al., 2018) |
|  | Intrusive questioning and showing mistrust (Wuyts et al., 2018) |
|  | Predominant parental Talking (Wuyts et al., 2018) |
| Guilt induction | Pressures other to agree (Allen et al., 1994; Mageau et al., 2016) |
|  | Showing disappointment and guilt inducing criticism (Barber, 1996; Mageau et al., 2016; Wuyts et al., 2018) |
| Invalidating feelings | Hostile or devaluing statement toward other (Allen et al., 1994) |
|  | Invalidating feelings (Barber, 1996) |
|  | Criticizing and expressing disapproval (Wuyts et al., 2018) |
| *Note.* Studies by Allen et al. (1994), Barber (1996), Hauser Kunz & Grych (2013), and Wuyts et al. (2018) included coding of parental autonomy support and/or psychological control. Study by Mageau et al. (2016) included child-reports of parental autonomy support. Love withdrawal is not included as it was difficult to observe in interaction tasks. | |

| **Supplementary Table 2**  *Bivariate Correlations of Main Variables per Group* | | | | | | | | | | | | | | | | | | |
| --- | --- | --- | --- | --- | --- | --- | --- | --- | --- | --- | --- | --- | --- | --- | --- | --- | --- | --- |
| Variable | 1. AS PSI | 2. AS EPI | 3. AS REM | 4. PC PSI | 5. PC EPI | 6. PC REM | 7. L/U PSI | 8. L/U EPI | 9. L/U REM | 10. C/D PSI | 11. C/D EPI | 12. C/D REM | 13. PA PSI | 14. PA EPI | 15. PA REM | 16. NA PSI | 17. NA EPI | 18. NA REM |
| 1. AS PSI |  | .31* | .46** | -.69** | -.16 | -.41** | .22 | .23 | .33** | -.02 | .10 | -.08 | .18 | .28* | .18 | .002 | -.10 | -.04 |
| 2. AS EPI | .17* |  | .16 | -.21 | -.67** | .02 | -.01 | .24 | .14 | -.03 | -.01 | .06 | -.01 | .09 | .11 | .10 | -.03 | -.06 |
| 3. AS REM | .31** | .24** |  | -.56** | -.10 | -.72** | .23 | .22 | .26* | -.12 | .20 | -.04 | .16 | .17 | .06 | -.07 | -.09 | -.10 |
| 4. PC PSI | -.69** | -.08 | -.13 |  | .08 | .58** | -.32* | -.36** | -.33** | .29* | .06 | .27* | -.19 | -.25 | -.13 | .08 | .14 | .06 |
| 5. PC EPI | -.02 | -.63** | -.18* | .11 |  | -.01 | .03 | -.09 | -.01 | .02 | -.01 | -.05 | .02 | -.001 | -.13 | -.02 | .01 | .09 |
| 6. PC REM | -.26** | -.27** | -.51** | .15 | .27** |  | -.37** | -.33** | -.41* | .25 | -.16 | .13 | -.42** | -.41** | -.41** | .13 | .13 | .20 |
| 7. L/U PSI | .09 | .12 | .16 | -.06 | .02 | -.04 |  | .75** | .73** | -.68** | -.49** | -.554** | .42** | .22 | .25* | -.62** | -.53** | -.51** |
| 8. L/U EPI | .01 | .16 | .05 | .00 | -.11 | -.06 | .56** |  | .79** | -.60** | -.60** | -.55** | .44** | .41** | .26* | -.53** | -.61** | -.56** |
| 9. L/U REM | -.05 | .08 | .12 | .10 | .06 | -.07 | .68** | .53** |  | -.47** | -.38** | -.62** | .32* | .25 | .31* | -.45** | -.44** | -.50** |
| 10. C/D PSI | -.13 | -.02 | -.05 | .06 | .04 | -.06 | -.51** | -.25** | -.25** |  | .73** | .56** | -.35** | -.09 | -.15 | .52** | .33** | .39** |
| 11. C/D EPI | .05 | -.06 | .08 | .01 | .18* | -.01 | -.22** | -.36** | -.23 | .48** |  | .59** | -.25 | -.05 | -.06 | .52** | .44** | .38** |
| 12. C/D REM | -.01 | -.003 | -.12 | -.08 | .06 | .07 | -.28** | -.21* | -.42** | .49** | .60** |  | -.24 | -.03 | -.12 | .57** | .34** | .40** |
| 13. PA PSI | .01 | -.05 | .02 | .02 | .10 | -.13 | .41** | .25** | .33** | -.28** | -.15 | -.16 |  | .79** | .74** | -.61** | -.58** | -.55** |
| 14. PA EPI | .002 | -.03 | .08 | .08 | .11 | -.03 | .32** | .27** | .34** | -.20* | -.16 | -.13 | .67** |  | .71** | -.30* | -.55** | -.41** |
| 15. PA REM | -.11 | -.10 | .13 | .13 | .12 | .08 | .28** | .36** | .33** | -.15 | -.22* | -.14 | .61** | .55** |  | -.34** | -.40** | -.53** |
| 16. NA PSI | .10 | .08 | -.05 | -.05 | -.06 | -.04 | -.52** | -.31** | -.49** | .25** | .23** | .32** | -.45** | -.30** | -.39** |  | .75** | .77** |
| 17. NA EPI | .13 | .03 | -.06 | -.06 | -.07 | -.03 | -.30** | -.21* | -.40** | .15 | .25** | .23** | -.27** | -.33** | -.39** | .62** |  | .75** |
| 18. NA REM | .15 | .06 | -.09 | -.09 | -.06 | -.08 | -.25** | -.29** | -.34** | .13 | .22** | .25** | -.33** | -.27** | -.57** | .53** | .63** |  |
| *Note.* AS = observed parental autonomy support; PC = observed parental psychological control; L/U = adolescent-perceived parental listening and understanding; C/D = adolescent-perceived parental criticism and dominance; PSI = problem solving interaction task; EPI = event planning interaction task; REM = reminiscence interaction task; PA = positive affect; NA = negative affect. Bivariate correlations of families with healthy control adolescent are presented below the diagonal, bivariate correlations families with an adolescent with current MDD/dysthymia are presented above the diagonal.  * *p* < .05; ** *p* < .01 | | | | | | | | | | | | | | | | | | |

| **Supplementary Table 3**  *Model Fit Statistics Multilevel Analyses Part 1 (H1.1-4)* | | | | | | | | |
| --- | --- | --- | --- | --- | --- | --- | --- | --- |
|  |  |  |  |  | Anova for model fit improvement | | | |
|  | Obs. | LL | AIC | BIC | Comparing | χ^2^ | df | *p* |
| Type of task > Observed AS |  |  |  |  |  |  |  |  |
| a. Unconditional model individual (model 1a) | 442 | -618.56 | 1243.1 | 1255.4 |  |  |  |  |
| b. Unconditional model individual and family (model 1b) | 442 | -614.32 | 1236.7 | 1253.0 | 1a and 1b | 8.48 | 1 | .004 |
| c. Type of task (EPI, REM) model (**model 2**) | 442 | -600.19 | 1212.4 | 1236.9 | 1b and 2 | 28.27 | 2 | <.001 |
| d. Type of task and random slope (EPI) model (model 3a) | 442 | -599.29 | 1218.6 | 1259.5 | 2 and 3a | 1.80 | 4 | .773 |
| e. Type of task and random slope (REM) model (model 3b) | 442 | -599.49 | 1219.0 | 1259.9 | 2 and 3b | 1.40 | 4 | .844 |
| f. Type of task and covariates model (model 4) | 442 | -599.23 | 1216.5 | 1253.3 | 2 and 4 | 1.92 | 3 | .589 |
| Type of task > Observed PC |  |  |  |  |  |  |  |  |
| a. Unconditional model individual (model 1a) | 442 | -623.67 | 1253.3 | 1265.6 |  |  |  |  |
| b. Unconditional model individual and family (model 1b) | 442 | -623.12 | 1254.2 | 1270.6 | 1a and 1b | 1.10 | 1 | .294 |
| c. Type of task (EPI, REM) model (**model 2**) | 442 | -606.81 | 1225.6 | 1250.2 | 1b and 2 | 32.63 | 2 | <.001 |
| d. Type of task and random slope (EPI) model (model 3a) | 442 | -604.84 | 1229.7 | 1270.6 | 2 and 3a | 3.925 | 4 | .416 |
| e. Type of task and random slope (REM) model (model 3b) | 442 | -606.81 | 1232.0 | 1272.9 | 2 and 3b | 1.603 | 4 | .808 |
| f. Type of task and covariates model (model 4) | 442 | -606.66 | 1231.3 | 1268.1 | 2 and 4 | 0.29 | 3 | .961 |
| Type task > L/U |  |  |  |  |  |  |  |  |
| a. Unconditional model individual (model 1a) | 437 | -571.52 | 1149.0 | 1161.3 |  |  |  |  |
| b. Unconditional model individual and family (model 1b) | 437 | -565.33 | 1138.7 | 1155.0 | 1a and 1b | 12.38 | 1 | <.001 |
| c. Type of task (EPI, REM) model (model 2) | 437 | -530.05 | 1072.1 | 1096.6 | 1b and 2 | 70.56 | 2 | <.001 |
| d. Type of task and random slope (EPI) model (model 3a) | 437 | -523.52 | 1067.0 | 1107.8 | 2 and 3a | 13.06 | 4 | .011 |
| e. Type of task and random slope (REM) model (model 3b) | 437 | -526.54 | 1073.1 | 1113.9 | 2 and 3b | 7.02 | 4 | .135 |
| f. Type of task, random slope (EPI), and all covariates model (model 4a)^a^ | 437 | -519.09 | 1064.2 | 1117.2 | 3a and 4a | 8.86 | 3 | .031 |
| g. Type of task, random slope (EPI), and covariate (biological sex parent) model (**model 4b**) | 437 | -520.33 | 1062.7 | 1107.5 | 3a and 4b | 6.38 | 1 | .012 |
| Type task > C/D |  |  |  |  |  |  |  |  |
| a. Unconditional model individual (model 1a) | 437 | -597.17 | 1200.3 | 1212.6 |  |  |  |  |
| b. Unconditional model individual and family (model 1b) | 437 | -583.13 | 1174.3 | 1190.6 | 1a and 1b | 28.08 | 1 | <.001 |
| c. Type of task (EPI, REM) model (model 2) | 437 | -513.84 | 1039.7 | 1064.2 | 1b and 2 | 138.6 | 2 | <.001 |
| d. Type of task and random slope (EPI) model (model 3a) | 437 | -512.45 | 1044.9 | 1085.7 | 2 and 3a | 2.78 | 4 | .596 |
| e. Type of task and random slope (REM) model (model 3b) | 437 | -513.46 | 1046.9 | 1087.7 | 2 and 3b | 0.75 | 4 | .945 |
| e. Type of task and covariates model (**model 4**) | 437 | -508.73 | 1035.5 | 1072.2 | 2 and 4 | 10.22 | 3 | .017 |
| AS -> L/U |  |  |  |  |  |  |  |  |
| a. Unconditional model individual (model 1a) | 437 | -571.52 | 1149.0 | 1161.3 |  |  |  |  |
| b. Unconditional model individual and family (model 1b) | 437 | -565.33 | 1138.7 | 1155.0 | 1a and 1b | 12.38 | 1 | <.001 |
| c. AS model (model 2) | 437 | -558.70 | 1127.4 | 1147.8 | 1b and 2 | 13.26 | 1 | <.001 |
| d. AS and random slope model (model 3) | 437 | -558.49 | 1135.0 | 1171.0 | 2 and 3 | 0.41 | 4 | .982 |
| e. AS and type of task (EPI, REM) model (model 4) | 437 | -528.20 | 1070.4 | 1099.0 | 2 and 4 | 61.00 | 2 | <.001 |
| f. AS*type of task model (model 5) | 437 | -527.63 | 1073.3 | 1110.0 | 4 and 5 | 1.14 | 2 | .567 |
| g. AS, type of task and covariates model (**model 6**) | 437 | -523.19 | 1066.4 | 1107.2 | 4 and 6 | 10.01 | 3 | .018 |
| AS -> C/D |  |  |  |  |  |  |  |  |
| a. Unconditional model individual (model 1a) | 437 | -597.17 | 1200.3 | 1212.6 |  |  |  |  |
| b. Unconditional model individual and family (model 1b) | 437 | -583.13 | 1174.3 | 1190.6 | 1a and 1b | 28.08 | 1 | <.001 |
| c. AS model (model 2) | 437 | -571.09 | 1152.2 | 1172.6 | 1b and 2 | 24.09 | 1 | <.001 |
| d. AS and random slope model (model 3) | 437 | -570.37 | 1158.8 | 1195.5 | 2 and 3 | 1.43 | 4 | .839 |
| e. AS and type of task (EPI, REM) model (model 4) | 437 | -508.80 | 1031.6 | 1060.2 | 2 and 4 | 124.58 | 2 | <.001 |
| f. AS*type of task model (model 5) | 437 | -508.56 | 1035.1 | 1071.8 | 4 and 5 | 0.47 | 2 | .789 |
| g. AS, type of task and covariates model (**model 6**) | 437 | -503.84 | 1027.7 | 1068.5 | 4 and 6 | 9.92 | 3 | .019 |
| PC -> L/U |  |  |  |  |  |  |  |  |
| a. Unconditional model individual (model 1a) | 437 | -571.52 | 1149.0 | 1161.3 |  |  |  |  |
| b. Unconditional model individual and family (model 1b) | 437 | -565.33 | 1138.7 | 1155.0 | 1a and 1b | 12.38 | 1 | <.001 |
| c. PC model (model 2) | 437 | -555.46 | 1120.9 | 1141.3 | 1b and 2 | 19.74 | 1 | <.001 |
| d. PC and random slope model (model 3) | 437 | -555.46 | 1128.6 | 1165.3 | 2 and 3 | 0.33 | 4 | .988 |
| e. PC and type of task (EPI, REM) model (model 4) | 437 | -527.15 | 1068.3 | 1096.9 | 2 and 4 | 56.62 | 2 | <.001 |
| f. PC*type of task model (model 5) | 437 | -526.31 | 1070.6 | 1107.3 | 4 and 5 | 1.67 | 2 | .434 |
| g. PC, type of task and covariates model (**model 6**) | 437 | -521.86 | 1063.7 | 1104.5 | 4 and 6 | 10.58 | 3 | .014 |
| PC -> C/D |  |  |  |  |  |  |  |  |
| a. Unconditional model individual (model 1a) | 437 | -597.17 | 1200.3 | 1212.6 |  |  |  |  |
| b. Unconditional model individual and family (model 1b) | 437 | -583.13 | 1174.3 | 1190.6 | 1a and 1b | 28.08 | 1 | <.001 |
| c. PC model (model 2) | 437 | -569.62 | 1149.2 | 1169.6 | 1b and 2 | 27.03 | 1 | <.001 |
| d. PC and random slope model (model 3) | 437 | -568.75 | 1155.5 | 1192.2 | 2 and 3 | 1.75 | 2 | .781 |
| e. PC and type of task (EPI, REM) model (model 4) | 437 | -509.91 | 1033.8 | 1062.4 | 2 and 4 | 119.43 | 2 | <.001 |
| f. PC*type of task model (model 5) | 437 | -509.14 | 1036.3 | 1073.0 | 4 and 5 | 1.54 | 2 | .464 |
| g. PC, type of task and covariates model (**model 6**) | 437 | -504.57 | 1029.2 | 1070.0 | 4 and 6 | 10.67 | 3 | .014 |
| AS -> Positive affect |  |  |  |  |  |  |  |  |
| a. Unconditional model individual (model 1a) | 437 | -570.16 | 1146.3 | 1158.6 |  |  |  |  |
| b. Unconditional model individual and family (model 1b) | 437 | -552.45 | 1112.9 | 1129.2 | 1a and 1b | 35.42 | 1 | <.001 |
| c. AS model (model 2) | 437 | -551.94 | 1113.9 | 1134.3 | 1b and 2 | 1.02 | 1 | .312 |
| d. AS and random slope model (model 3) | 437 | -551.79 | 1121.6 | 1158.3 | 2 and 3 | 0.31 | 4 | .989 |
| e. AS and type of task (EPI, REM) model (model 4) | 437 | -511.35 | 1036.7 | 1065.2 | 2 and 4 | 81.19 | 2 | <.001 |
| f. AS*type of task model (model 5) | 437 | -506.35 | 1030.7 | 1067.4 | 4 and 5 | 9.99 | 2 | .007 |
| g. AS*type of task, and pre-task affect model (**model 6**)^a^ | 434 | -502.51 | 1025.0 | 1065.7 | 5 and 6 | 1.875 | 1 | .171 |
| h. AS*type of task, pre-task affect, and covariates model (model 7) | 434 | -500.47 | 1026.9 | 1079.9 | 5 and 6 | 4.071 | 3 | .254 |
| AS -> Negative affect |  |  |  |  |  |  |  |  |
| a. Unconditional model individual (model 1a) | 437 | -581.27 | 1168.5 | 1180.8 |  |  |  |  |
| b. Unconditional model individual and family (model 1b) | 437 | -560.98 | 1130.0 | 1146.3 | 1a and 1b | 40.57 | 1 | <.001 |
| c. AS model (model 2) | 437 | -560.94 | 1131.9 | 1152.3 | 1b and 2 | 0.07 | 1 | .785 |
| d. AS and random slope model (model 3) | 437 | -560.25 | 1138.5 | 1175.2 | 2 and 3 | 1.39 | 4 | .845 |
| e. AS and type of task (EPI, REM) model (**model 4**) | 437 | -534.63 | 1083.2 | 1111.8 | 2 and 4 | 52.63 | 2 | <.001 |
| f. AS*type of task model (model 5) | 437 | -534.02 | 1086.0 | 1122.8 | 4 and 5 | 1.21 | 2 | .545 |
| g. AS, type of task, and pre-task affect model (model 6)^a^ | 434 | -528.25 | 1072.5 | 1105.1 | 4 and 6 | 2.637 | 1 | .104 |
| g. AS, type of task, pre-task affect, and covariates model (model 7) | 434 | -524.49 | 1071.0 | 1115.8 | 6 and 7 | 7.525 | 3 | .057 |
| PC -> Positive affect |  |  |  |  |  |  |  |  |
| a. Unconditional model individual (model 1a) | 437 | -570.16 | 1146.3 | 1158.6 |  |  |  |  |
| b. Unconditional model individual and family (model 1b) | 437 | -552.45 | 1112.9 | 1129.2 | 1a and 1b | 35.42 | 1 | <.001 |
| c. PC model (model 2) | 437 | -552.36 | 1114.7 | 1135.1 | 1b and 2 | 0.17 | 1 | .674 |
| d. PC and random slope model (model 3) | 437 | -547.47 | 1113.0 | 1149.7 | 2 and 3 | 9.78 | 4 | .044 |
| e. PC, random slope and type of task (EPI, REM) model (model 4) | 437 | -504.85 | 1031.7 | 1076.6 | 3 and 4 | 85.24 | 2 | <.001 |
| f. PC*type of task and random slope model (model 5) | 437 | -502.22 | 1030.4 | 1083.5 | 4 and 5 | 5.27 | 2 | .072 |
| g. PC, random slope, type of task, and pre-task affect model (**model 6**)^b^ | 434 | -498.77 | 1021.5 | 1070.4 | 4 and 6 | 5.320 | 1 | .021 |
| g. PC, random slope, type of task, pre-task affect, and covariates model (model 7) | 434 | -496.52 | 1023.0 | 1084.1 | 6 and 7 | 4.499 | 3 | .212 |
| PC -> Negative affect |  |  |  |  |  |  |  |  |
| a. Unconditional model individual (model 1a) | 437 | -581.27 | 1168.5 | 1180.8 |  |  |  |  |
| b. Unconditional model individual and family (model 1b) | 437 | -560.98 | 1130.0 | 1146.3 | 1a and 1b | 40.57 | 1 | <.001 |
| c. PC model (model 2) | 437 | -560.98 | 1132.0 | 1152.4 | 1b and 2 | 0.0002 | 1 | .988 |
| d. PC and random slope model (model 3) | 437 | -560.50 | 1139.0 | 1175.7 | 2 and 3 | 0.961 | 4 | .916 |
| e. PC and type of task (EPI, REM) model (model 4) | 437 | -534.77 | 1083.5 | 1112.1 | 2 and 4 | 52.43 | 2 | <.001 |
| f. PC*type of task model (model 5) | 437 | -534.27 | 1086.5 | 1123.2 | 4 and 5 | 1.004 | 2 | .605 |
| g. PC, type of task, and pre-task affect model (model 6)^b^ | 434 | -528.35 | 1072.7 | 1105.3 | 4 and 6 | 2.616 | 1 | .106 |
| h. PC, type of task, pre-task affect, and covariates model (**model 7)** | 434 | -524.51 | 1071.0 | 1115.8 | 6 and 7 | 7.685 | 3 | .053 |
| AS -> L/U daily life |  |  |  |  |  |  |  |  |
| a. Unconditional model family (model 1) | 142 | -185.40 | 376.80 | 385.67 |  |  |  |  |
| b. AS model (**model 2**) | 142 | -184.29 | 376.57 | 388.40 | 1 and 2 | 2.227 | 1 | .136 |
| c. AS and covariates model (model 3) | 142 | -182.12 | 378.24 | 398.93 | 2 and 3 | 4.330 | 3 | .228 |
| AS -> C/D daily life |  |  |  |  |  |  |  |  |
| a. Unconditional model family (model 1) | 142 | -187.47 | 380.95 | 389.82 |  |  |  |  |
| b. AS model (**model 2**) | 142 | -187.26 | 382.53 | 394.35 | 1 and 2 | 0.422 | 1 | .516 |
| c. AS and covariates model (model 3) | 142 | -185.66 | 385.31 | 406.00 | 2 and 3 | 3.215 | 3 | .360 |
| PC -> L/U daily life |  |  |  |  |  |  |  |  |
| a. Unconditional model family (model 1) | 142 | -185.40 | 376.80 | 385.67 |  |  |  |  |
| b. PC model (**model 2**) | 142 | -182.35 | 372.69 | 384.52 | 1 and 2 | 6.107 | 1 | .013 |
| c. PC and covariates model (model 3) | 142 | -179.72 | 373.43 | 394.13 | 2 and 3 | 5.258 | 3 | .154 |
| PC -> C/D daily life |  |  |  |  |  |  |  |  |
| a. Unconditional model family (model 1) | 142 | -187.47 | 380.95 | 389.82 |  |  |  |  |
| b. PC model (**model 2**) | 142 | -186.85 | 381.71 | 393.53 | 1 and 2 | 1.239 | 1 | .266 |
| c. PC and covariates model (model 3) | 142 | -185.20 | 384.39 | 405.09 | 2 and 3 | 3.315 | 3 | .346 |
| *Note.* AS = parental autonomy support; PC = parental psychological control; L/U = parental listening and understanding; C/D parental criticism and dominance; PSI = problem solving interaction task; EPI = event planning interaction task; REM = reminiscence interaction task. Covariates: parental sex, adolescent sex and age. Sex: 0 = female, 1 = male. Type of task model: dummy coding with PSI as reference category.  ^a^ Model did run, but an error appeared warning for the complexity of the model given the number of parameters relative to the number of observations. We decided to run the model again, only including the significant covariate, and dropping other covariates to enhance simplicity and thereby stability of the model.  ^b^ Model fit comparison could not run on model 5 and 6 with adolescent affect as the dependent variable, because of missing data for pre-task affect (*n* = 3 observations). In order to compute model fit statistics in comparing model 5 and 6 with adolescent affect as the dependent variable, cases with missing data for pre-task affect were excluded in the comparison of these models. | | | | | | | | |

| **Supplementary Table 4**  *Results Multilevel Analyses on the Effect of Type of Task on Observed AS and PC (H1.1), and Perceived L/U and C/D (H1.2)* | | | | | | | | | | |
| --- | --- | --- | --- | --- | --- | --- | --- | --- | --- | --- |
|  |  |  | Observed AS | | Observed PC | | Perceived L/U | | Perceived C/D | |
|  |  |  | Estimate (*SE*) | *p* | Estimate (*SE*) | *p* | Estimate (*SE*) | *p* | Estimate (*SE*) | *p* |
| Fixed effects | | |  |  |  |  |  |  |  |  |
|  | Intercept | | -0.256 (0.086) | .003 | 0.345 (0.082) | <.001 | -0.500 (0.106) | <.001 | 0.388 (0.898) | .667 |
|  | Type of task | |  |  |  |  |  |  |  |  |
|  |  | EPI v. PSI | 0.262 (0.099) | .009 | -0.543 (0.103) | <.001 | 0.566 (0.075) | <.001 | -0.836 (0.074) | <.001 |
|  |  | REM v. PSI | 0.541 (0.099) | <.001 | -0.504 (0.103) | <.001 | 0.578 (0.072) | <.001 | -0.885 (0.075) | <.001 |
|  |  | REM v. EPI^a^ | 0.279 (0.099) | .005 | 0.039 (0.103) | .708 | 0.010 (0.069) | .924 | -0.049 (0.075) | .513 |
|  | Biological sex parent | |  |  |  |  | 0.256 (0.097) | .010 | -0.188 (0.079) | .021 |
|  | Biological sex adolescent | |  |  |  |  |  |  | -0.355 (0.160) | .029 |
|  | Age adolescent | |  |  |  |  |  |  | 0.032 (0.057) | .582 |
| Random effects | | |  |  |  |  |  |  |  |  |
|  | Between person variance | | 0.060 |  | 0.099 |  | 0.328 |  | 0.084 |  |
|  | Within person variance | | 0.724 |  | 0.785 |  | 0.371 |  | 0.405 |  |
|  | Between family variance | | 0.168 |  | 0.052 |  | 0.289 |  | 0.312 |  |
|  | Random effect variance person | |  |  |  |  | 0.073 |  |  |  |
|  | Random effect variance family | |  |  |  |  | 0.005 |  |  |  |
| Intraclass correlation | | |  |  |  |  |  |  |  |  |
|  | Person level | | .04 |  | .05 |  | .23 |  | .02 |  |
|  | Family level | | .17 |  | .07 |  | .26 |  | .32 |  |
| *Note.* *N* = 148 parents; *n* = 442 observations of observed parenting; *n* = 437 observations of perceived parenting. AS = parental autonomy support; PC = parental psychological control; L/U = parental listening and understanding; C/D = parental criticism and dominance; HC = families with a healthy control adolescent; DEP = families with an adolescent with current MDD/dysthymia; PSI = problem solving interaction task; EPI = event planning interaction task; REM = reminiscence interaction task. Biological sex: 0 = female, 1 = male.  ^a^ Tested in a separate model. Output for this variable is based on the model including EPI as the reference category in dummy coding. Whereas all other output is based on the model including PSI as the reference category in dummy coding. | | | | | | | | | | |

| **Supplementary Table 5**  *Results Multilevel Analyses on the Relation between Observed AS and PC, and Perceived L/U and C/D (H1.3)* | | | | | | | | | | |
| --- | --- | --- | --- | --- | --- | --- | --- | --- | --- | --- |
|  |  |  | Observed AS | | | | Observed PC | | | |
|  |  |  | Perceived L/U | | Perceived criticism | | Perceived L/U | | Perceived C/D | |
|  |  |  | Estimate (*SE*) | *p* | Estimate (*SE*) | *p* | Estimate (*SE*) | *p* | Estimate (*SE*) | *p* |
| Fixed effects | | |  |  |  |  |  |  |  |  |
|  | Intercept | | 0.491 (0.924) | .597 | 0.420 (0.896) | .640 | 0.516 (0.931) | .580 | 0.383 (0.897) | .670 |
|  | Observed parenting | | 0.072 (0.039) | .067 | -0.120 (0.038) | .002 | -0.093 (0.038) | .014 | 0.107 (0.037) | .004 |
|  | Type of task | |  |  |  |  |  |  |  |  |
|  |  | EPI v. PSI | 0.548 (0.074) | <.001 | -0.804 (0.074) | <.001 | 0.517 (0.076) | <.001 | -0.779 (0.076) | <.001 |
|  |  | REM v. PSI | 0.537 (0.077) | <.001 | -0.821 (0.076) | <.001 | 0.529 (0.076) | <.001 | -0.831 (0.076) | <.001 |
|  |  | REM v. EPI^a^ | -0.011 (0.075) | .883 | -0.016 (0.075) | .830 | 0.012 (0.074) | .871 | -0.053 (0.074) | .478 |
|  | Biological sex parent | | 0.300 (0.100) | .004 | -0.177 (0.079) | .029 | 0.310 (0.100) | .003 | -0.192 (0.079) | .017 |
|  | Biological sex adolescent | | 0.048 (0.163) | .768 | -0.364 (0.159) | .025 | 0.045 (0.164) | .786 | -0.357 (0.159) | .028 |
|  | Age adolescent | | -0.065 (0.059) | .275 | 0.028 (0.057) | .628 | -0.066 (0.059) | .272 | 0.030 (0.057) | .602 |
| Random effects | | |  |  |  |  |  |  |  |  |
|  | Between person variance | | 0.219 |  | 0.088 |  | 0.221 |  | 0.082 |  |
|  | Within person variance | | 0.396 |  | 0.392 |  | 0.391 |  | 0.396 |  |
|  | Between family variance | | 0.260 |  | 0.310 |  | 0.267 |  | 0.313 |  |
| Intraclass correlation | | |  |  |  |  |  |  |  |  |
|  | Person level | | .23 |  | .02 |  | .23 |  | .02 |  |
|  | Family level | | .26 |  | .32 |  | .26 |  | .32 |  |
| *Note.* *N* = 148 parents; *n* = 442 observations of observed parenting; *n* = 437 observations of perceived parenting. AS = parental autonomy support; PC = parental psychological control; L/U = parental listening and understanding; C/D = parental criticism and dominance; HC = families with a healthy control adolescent; DEP = families with an adolescent with current MDD/dysthymia; PSI = problem solving interaction task; EPI = event planning interaction task; REM = reminiscence interaction task. Biological sex: 0 = female, 1 = male.  ^a^ Tested in a separate model. Output for this variable is based on the model including EPI as the reference category in dummy coding. Whereas all other output is based on the model including PSI as the reference category in dummy coding. | | | | | | | | | | |

| **Supplementary Table 6**  *Results Multilevel Analyses on the Relation between Observed AS and PC, and Adolescent Positive and Negative Affect (H1.3)* | | | | | | | | | | |
| --- | --- | --- | --- | --- | --- | --- | --- | --- | --- | --- |
|  |  |  | Observed AS | | | | Observed PC | | | |
|  |  |  | Positive affect | | Negative affect | | Positive affect | | Negative affect | |
|  |  |  | Estimate (*SE*) | *p* | Estimate (*SE*) | *p* | Estimate (*SE*) | *p* | Estimate (*SE*) | *p* |
| Fixed effects | | |  |  |  |  |  |  |  |  |
|  | Intercept | | -0.061 (.089) | .498 | 0.002 (.088) | .985 | -0.018 (.084) | .829 | 0.009 (.089) | .918 |
|  | Observed parenting | | 0.054 (.060) | .368 | -0.020 (.041) | .629 | 0.036 (.046) | .440 | -0.008 (.040) | .834 |
|  | Type of task | |  |  |  |  |  |  |  |  |
|  |  | EPI v. PSI | 0.467 (.076) | <.001 | -0.331 (.082) | <.001 | 0.469 (.078) | <.001 | -0.340 (.084) | <.001 |
|  |  | REM v. PSI | -0.222 (.086) | .010 | -0.330 (.087) | <.001 | -0.307 (.086) | <.001 | 0.315 (.087) | <.001 |
|  |  | REM v. EPI^a^ | -0.690 (.080) | <.001 | 0.661 (.085) | <.001 | -0.776 (.079) | <.001 | 0.656 (.084) | <.001 |
|  | Observed AS/PC*Type of task | |  |  |  |  |  |  |  |  |
|  |  | Observed*EPI (v. PSI) | -0.008 (.082) | .926 |  |  |  |  |  |  |
|  |  | Observed*REM (v. PSI) | -0.224 (.082) | .006 |  |  |  |  |  |  |
|  |  | Observed*REM (v. EPI) ^a^ | -0.216 (.084) | .011 |  |  |  |  |  |  |
|  | Pre-task affect | | 0.101 (.047) | .032 | 0.095 (.045) | .038 | 0.169 (.046) | <.001 | 0.095 (.045) | .037 |
| Random effects | | |  |  |  |  |  |  |  |  |
|  | Between person variance | | .084 |  | .039 |  | .032 |  | .039 |  |
|  | Within person variance | | .393 |  | .485 |  | .389 |  | .486 |  |
|  | Between family variance | | .336 |  | .323 |  | .277 |  | .319 |  |
|  | Random effect variance person | |  |  |  |  | .009 |  |  |  |
|  | Random effect variance family | |  |  |  |  | .050 |  |  |  |
| Intraclass correlation | | |  |  |  |  |  |  |  |  |
|  | Person level | | .07 |  | .03 |  | .07 |  | .03 |  |
|  | Family level | | .42 |  | .40 |  | .42 |  | .40 |  |
| *Note.* *N* = 148 parents; *n* = 442 observations of observed parenting; *n* = 437 observations of perceived parenting. AS = parental autonomy support; PC = parental psychological control; L/U = parental listening and understanding; C/D = parental criticism and dominance; HC = families with a healthy control adolescent; DEP = families with an adolescent with current MDD/dysthymia; PSI = problem solving interaction task; EPI = event planning interaction task; REM = reminiscence interaction task. Biological sex: 0 = female, 1 = male.  ^a^ Tested in a separate model. Output for this variable is based on the model including EPI as the reference category in dummy coding. Whereas all other output is based on the model including PSI as the reference category in dummy coding. | | | | | | | | | | |

| **Supplementary Table 7**  *Results Multilevel Analyses on the Relation between Observed AS and PC, and Perceived L/U and C/D in Daily Life (H1.4)* | | | | | | | | | | |
| --- | --- | --- | --- | --- | --- | --- | --- | --- | --- | --- |
|  |  |  | Observed AS | | | | Observed PC | | | |
|  |  |  | EMA L/U | | EMA Criticism | | EMA L/U | | EMA C/D | |
|  |  |  | Estimate (*SE*) | *p* | Estimate (*SE*) | *p* | Estimate (*SE*) | *p* | Estimate (*SE*) | *p* |
| Fixed effects | | |  |  |  |  |  |  |  |  |
|  | Intercept | | -0.001 (.103) | .991 | 0.014 (.102) | .894 | -0.001 (.105) | .995 | 0.014 (.102) | .894 |
|  | Observed parenting | | 0.114 (.076) | .136 | -0.051 (.078) | .517 | -0.176 (.068) | .011 | 0.081 (.073) | .266 |
| Random effects | | |  |  |  |  |  |  |  |  |
|  | Within person variance | | .359 |  | .409 |  | .322 |  | .581 |  |
|  | Between family variance | | .631 |  | .576 |  | .683 |  | .403 |  |
| Intraclass correlation | | |  |  |  |  |  |  |  |  |
|  | Family level | | .63 |  | .59 |  | .63 |  | .59 |  |
| *Note.* *N* = 148 parents; *n* = 148 observations of observed parenting; *n* = 142 observations of perceived parenting in daily life. AS = parental autonomy support; PC = parental psychological control; L/U = parental listening and understanding; C/D = parental criticism and dominance; HC = families with a healthy control adolescent; DEP = families with an adolescent with current MDD/dysthymia; PSI = problem solving interaction task; EPI = event planning interaction task; REM = reminiscence interaction task. Biological sex: 0 = female, 1 = male. | | | | | | | | | | |

| **Supplementary Table 8**  *Model Fit Statistics Multilevel Analyses Part 2 (H2.1-3)* | | | | | | | | |
| --- | --- | --- | --- | --- | --- | --- | --- | --- |
|  |  |  |  |  | Anova for model fit improvement | | | |
|  | Obs. | LL | AIC | BIC | Comparing | χ^2^ | Df | *p* |
| Group -> Observed AS |  |  |  |  |  |  |  |  |
| a. Unconditional model individual (model 1a) | 628 | -874.78 | 1755.6 | 1768.9 |  |  |  |  |
| b. Unconditional model individual and family (model 1b) | 628 | -870.89 | 1749.8 | 1767.5 | 1a and 1b | 7.777 | 1 | .005 |
| c. Group (HC v. DEP) model (model 2) | 628 | -869.69 | 1749.4 | 1771.6 | 1b and 2 | 2.397 | 1 | .122 |
| d. Group and random slope model (model 3) | 628 | -867.33 | 1752.7 | 1792.6 | 2 and 3 | 4.732 | 4 | .316 |
| e. Group and type of task (EPI, REM) model (**model 4**) | 628 | -852.77 | 1719.5 | 1750.6 | 2 and 4 | 33.836 | 2 | <.001 |
| f. Group*type of task model (model 5) | 628 | -852.54 | 1723.1 | 1763.1 | 4 and 5 | 0.470 | 2 | .791 |
| f. Group, type of task and covariates model (model 6) | 628 | -850.39 | 1720.8 | 1765.2 | 4 and 6 | 4.773 | 3 | .189 |
| Goup -> Observed PC |  |  |  |  |  |  |  |  |
| a. Unconditional model individual (model 1a) | 628 | -883.28 | 1772.6 | 1785.9 |  |  |  |  |
| b. Unconditional model individual and family (model 1b) | 628 | -883.28 | 1774.6 | 1792.3 | 1a and 1b | 0.001 | 1 | .975 |
| c. Group (HC v. DEP) model (model 2) | 628 | -882.95 | 1775.9 | 1798.1 | 1b and 2 | 0.672 | 1 | .412 |
| d. Group and random slope model (model 3) | 628 | -880.28 | 1778.6 | 1818.5 | 2 and 3 | 5.324 | 4 | .256 |
| e. Group and type of task (EPI, REM) model (**model 4**) | 628 | -865.29 | 1744.6 | 1775.7 | 2 and 4 | 35.316 | 2 | <.001 |
| f. Group*type of task model (model 5) | 628 | -864.73 | 1747.5 | 1787.5 | 4 and 5 | 1.108 | 2 | .575 |
| g. Group, type of task covariates model (model 6) | 628 | -865.17 | 1750.3 | 1794.8 | 4 and 6 | 0.230 | 3 | .973 |
| Group -> L/U |  |  |  |  |  |  |  |  |
| a. Unconditional model individual (model 1a) | 622 | -779.07 | 1564.2 | 1577.5 |  |  |  |  |
| b. Unconditional model individual and family (model 1b) | 622 | -768.74 | 1545.5 | 1563.2 | 1a and 1b | 20.673 | 1 | <.001 |
| c. Group (HC v. DEP) model (model 2) | 622 | -764.58 | 1539.2 | 1561.3 | 1b and 2 | 8.314 | 1 | .004 |
| d. Group and random slope model (model 3) | 622 | -756.35 | 1530.7 | 1570.6 | 2 and 3 | 16.54 | 4 | .002 |
| e. Group and type of task (EPI, REM) model (**model 4**) | 622 | -715.44 | 1444.9 | 1475.9 | 2 and 4 | 98.274 | 2 | <.001 |
| f. Group*type of task model (model 5) | 622 | -714.39 | 1446.8 | 1486.7 | 4 and 5 | 2.113 | 2 | .348 |
| g. Group, random slope, type of task, and covariates model (model 6) | 622 | -712.81 | 1445.6 | 1490.0 | 4 and 6 | 5.262 | 3 | .154 |
| Group -> C/D |  |  |  |  |  |  |  |  |
| a. Unconditional model individual (model 1a) | 622 | -832.92 | 1671.8 | 1685.1 |  |  |  |  |
| b. Unconditional model individual and family (model 1b) | 622 | -811.66 | 1631.3 | 1649.0 | 1a and 1b | 42.519 | 1 | <.001 |
| c. Group (HC v. DEP) model (model 2) | 622 | -810.48 | 1631.0 | 1653.1 | 1b and 2 | 2.361 | 1 | .124 |
| d. Group and random slope model (model 3) | 622 | -807.00 | 1632.0 | 1671.9 | 2 and 3 | 6.957 | 4 | .138 |
| e. Group and type of task model (model 4) | 622 | -727.78 | 1469.6 | 1500.6 | 2 and 4 | 165.390 | 2 | <.001 |
| f. Group*type of task model (model 5) | 622 | -725.09 | 1468.2 | 1508.1 | 4 and 5 | 5.398 | 2 | .067 |
| g. Group*type of task and covariates model (**model 6**) | 622 | -720.49 | 1465.0 | 1518.2 | 5 and 6 | 9.193 | 3 | .027 |
| Observed AS -> L/U |  |  |  |  |  |  |  |  |
| a. Unconditional model individual (model 1a) | 622 | -779.07 | 1564.2 | 1577.5 |  |  |  |  |
| b. Unconditional model individual and family (model 1b) | 622 | -768.74 | 1545.5 | 1563.2 | 1a and 1b | 20.673 | 1 | <.001 |
| c. AS model (model 2) | 622 | -757.87 | 1525.8 | 1547.9 | 1b and 2 | 21.730 | 1 | <.001 |
| d. AS and random slope model (model 3) | 622 | -755.34 | 1528.7 | 1568.6 | 2 and 3 | 5.068 | 4 | .280 |
| e. AS and group (HC v. DEP) model (model 4) | 622 | -754.05 | 1520.1 | 1546.7 | 3 and 4 | 7.653 | 1 | .006 |
| f. AS*group model (model 5) | 622 | -753.89 | 1521.8 | 1552.8 | 4 and 5 | 0.320 | 1 | .572 |
| g. AS*group and type of task (EPI, REM) model (**model 6**) | 622 | -710.69 | 1439.4 | 1479.3 | 5 and 6 | 86.385 | 2 | <.001 |
| h. AS*group, type of task (EPI, REM), and covariate model (model 7) | 622 | -708.39 | 1440.8 | 1494.0 | 6 and 7 | 4.602 | 3 | .203 |
| Observed AS -> C/D |  |  |  |  |  |  |  |  |
| a. Unconditional model individual (model 1a) | 622 | -832.92 | 1671.8 | 1685.1 |  |  |  |  |
| b. Unconditional model individual and family (model 1b) | 622 | -811.66 | 1631.3 | 1649.0 | 1a and 1b | 42.519 | 1 | <.001 |
| c. AS model (model 2) | 622 | -798.98 | 1608.0 | 1630.1 | 1b and 2 | 25.354 | 1 | <.001 |
| d. AS and random slope model (model 3) | 622 | -798.19 | 1614.4 | 1654.3 | 2 and 3 | 1.585 | 4 | .811 |
| e. AS and group (HC v. DEP) model (model 4) | 622 | -798.13 | 1608.3 | 1634.9 | 2 and 4 | 1.701 | 1 | .192 |
| f. AS*group model (model 5) | 622 | -796.99 | 1608.0 | 1639.0 | 4 and 5 | 2.292 | 1 | .130 |
| g. AS*group and type of task (EPI, REM) model (model 6) | 622 | -721.69 | 1461.4 | 1501.3 | 5 and 6 | 150.59 | 2 | <.001 |
| h. AS*group, type of task, and covariates model (**model 7**) | 622 | -717.25 | 1458.5 | 1511.7 | 6 and 7 | 8.882 | 3 | .031 |
| Observed PC -> L/U |  |  |  |  |  |  |  |  |
| a. Unconditional model individual (model 1a) | 622 | -779.07 | 1564.2 | 1577.5 |  |  |  |  |
| b. Unconditional model individual and family (model 1b) | 622 | -768.74 | 1545.5 | 1563.2 | 1a and 1b | 20.673 | 1 | <.001 |
| c. PC model (model 2) | 622 | -752.34 | 1514.7 | 1536.8 | 1b and 2 | 32.803 | 1 | <.001 |
| d. PC and random slope model (model 3) | 622 | -752.07 | 1522.1 | 1562.0 | 2 and 3 | 0.531 | 4 | .970 |
| e. PC and group (HC v. DEP) model (model 4) | 622 | -748.31 | 1508.6 | 1535.2 | 2 and 4 | 8.047 | 1 | .005 |
| f. PC*group model (model 5) | 622 | -748.15 | 1510.3 | 1541.3 | 4 and 5 | 0.326 | 1 | .568 |
| g. PC*group and type of task (EPI, REM) model (**model 6**) | 622 | -708.49 | 1435.0 | 1474.9 | 5 and 6 | 79.323 | 2 | <.001 |
| h. PC*group, type of task, and covariates model (model 7) | 622 | -705.85 | 1435.7 | 1488.9 | 6 and 7 | 5.268 | 3 | .153 |
| Observed PC -> C/D |  |  |  |  |  |  |  |  |
| a. Unconditional model individual (model 1a) | 622 | -832.92 | 1671.8 | 1685.1 |  |  |  |  |
| b. Unconditional model individual and family (model 1b) | 622 | -811.66 | 1631.3 | 1649.0 | 1a and 1b | 42.519 | 1 | <.001 |
| c. PC model (model 2) | 622 | -793.08 | 1596.2 | 1618.3 | 1b and 2 | 37.169 | 1 | <.001 |
| d. PC and random slope model (model 3) | 622 | -792.31 | 1602.6 | 1642.5 | 2 and 3 | 1.527 | 4 | .822 |
| e. PC and group (HC v. DEP) model (model 4) | 622 | -792.08 | 1596.2 | 1622.8 | 2 and 4 | 1.998 | 1 | .158 |
| f. PC*group model (model 5) | 622 | -791.93 | 1597.9 | 1628.9 | 4 and 5 | 0.299 | 1 | .584 |
| g. PC*group and type of task (EPI, REM) model (model 6) | 622 | -720.65 | 1459.3 | 1499.2 | 5 and 6 | 142.56 | 2 | <.001 |
| i. PC*group, type of task, and covariates model (**model 7**) | 622 | -715.92 | 1455.8 | 1509.0 | 6 and 7 | 9.462 | 3 | .024 |
| Observed AS -> Positive affect |  |  |  |  |  |  |  |  |
| a. Unconditional model individual (model 1a) | 623 | -732.38 | 1470.8 | 1484.1 |  |  |  |  |
| b. Unconditional model individual and family (model 1b) | 623 | -685.57 | 1379.1 | 1396.9 | 1a and 1b | 93.621 | 1 | <.001 |
| c. AS model (model 2) | 623 | -685.33 | 1380.7 | 1402.8 | 1b and 2 | 0.470 | 1 | .493 |
| d. AS and random slope model (model 3) | 623 | -682.96 | 1383.9 | 1423.8 | 2 and 3 | 4.740 | 4 | .315 |
| e. AS and group (HC v. DEP) model (model 4) | 623 | -657.67 | 1327.3 | 1354.0 | 2 and 4 | 55.318 | 1 | <.001 |
| f. AS*group model (model 5) | 623 | -657.56 | 1329.1 | 1360.2 | 4 and 5 | 0.215 | 1 | .643 |
| g. AS*group and type of task (EPI, REM) model (model 6) | 623 | -595.93 | 1209.9 | 1249.8 | 5 and 6 | 123.260 | 2 | <.001 |
| h. AS*group, type of task, and pre-task affect model (**model 7**)^a^ | 620 | -590.91 | 1201.8 | 1246.1 | 6 and 7 | 5.63 | 1 | .018 |
| i. AS*condition, type of task, pre-task affect, and covariates model (model 8) | 620 | -589.30 | 1204.6 | 1262.2 | 7 and 8 | 3.226 | 3 | .358 |
| Observed AS -> Negative affect |  |  |  |  |  |  |  |  |
| a. Unconditional model individual (model 1a) | 623 | -703.03 | 1412.1 | 1425.4 |  |  |  |  |
| b. Unconditional model individual and family (model 1b) | 623 | -648.66 | 1305.3 | 1323.1 | 1a and 1b | 108.74 | 1 | <.001 |
| c. PC model (model 2) | 623 | -648.61 | 1307.2 | 1329.4 | 1b and 2 | 0.108 | 1 | .743 |
| d. PC and random slope model (model 3) | 623 | -645.99 | 1310.0 | 1349.9 | 2 and 3 | 5.251 | 4 | .263 |
| e. PC and group (HC v. DEP) model (model 4) | 623 | -620.55 | 1253.1 | 1279.7 | 2 and 4 | 56.12 | 1 | <.001 |
| f. PC*group model (model 5) | 623 | -620.47 | 1254.9 | 1286.0 | 4 and 5 | 0.165 | 1 | .685 |
| g. PC*group and type of task (EPI, REM) model (model 6) | 623 | -584.54 | 1187.1 | 1227.0 | 5 and 6 | 71.859 | 2 | <.001 |
| h. PC*group, type of task, and pre-task affect model (**model 7**)^a^ | 620 | -576.65 | 1173.3 | 1217.6 | 6 and 7 | 9.987 | 1 | .002 |
| f. PC*group, type of task, pre-task affect, and covariates model (model 7) | 620 | -576.41 | 1178.8 | 1236.4 | 7 and 8 | 0.480 | 3 | .923 |
| Observed PC -> Positive affect |  |  |  |  |  |  |  |  |
| a. Unconditional model individual (model 1a) | 623 | -732.38 | 1470.8 | 1484.1 |  |  |  |  |
| b. Unconditional model individual and family (model 1b) | 623 | -685.57 | 1379.1 | 1396.9 | 1a and 1b | 93.621 | 1 | <.001 |
| c. PC model (model 2) | 623 | -684.47 | 1378.9 | 1401.1 | 1b and 2 | 2.200 | 1 | .138 |
| d. PC and random slope model (model 3) | 623 | -680.47 | 1378.9 | 1418.8 | 2 and 3 | 7.998 | 4 | .092 |
| e. PC and group (HC v. DEP) model (model 4) | 623 | -656.97 | 1325.9 | 1352.5 | 2 and 4 | 54.989 | 1 | <.001 |
| f. PC*group model (model 5) | 623 | -656.30 | 1326.6 | 1357.6 | 4 and 5 | 1.338 | 1 | .247 |
| g. PC*group and type of task (EPI, REM) model (model 6) | 623 | -595.30 | 1208.6 | 1248.5 | 5 and 6 | 122.000 | 2 | <.001 |
| h. PC*group, type of task, and pre-task affect model (**model 7**)^a^ | 620 | -592.78 | 1205.6 | 1249.9 | 6 and 7 | 0.784 | 1 | .376 |
| h. PC*group, type of task, pre-task affect, and covariates model (model 8) | 620 | -591.42 | 1208.8 | 1266.4 | 7 and 8 | 2.728 | 3 | .436 |
| Observed PC -> Negative affect | 623 |  |  |  |  |  |  |  |
| a. Unconditional model individual (model 1a) | 623 | -703.03 | 1412.1 | 1425.4 |  |  |  |  |
| b. Unconditional model individual and family (model 1b) | 623 | -648.66 | 1305.3 | 1323.1 | 1a and 1b | 108.74 | 1 | <.001 |
| c. PC model (model 2) | 623 | -647.35 | 1304.7 | 1326.9 | 1b and 2 | 2.622 | 1 | .105 |
| d. PC and random slope model (model 3) | 623 | -645.54 | 1309.1 | 1349.0 | 2 and 3 | 3.630 | 4 | .458 |
| e. PC and group (HC v. DEP) model (model 4) | 623 | -619.44 | 1250.9 | 1277.5 | 2 and 4 | 55.828 | 1 | <.001 |
| f. PC*group model (model 5) | 623 | -617.68 | 1249.4 | 1280.4 | 4 and 5 | 3.515 | 1 | .061 |
| g. PC*group and type of task model (EPI, REM) (model 6) | 623 | -582.47 | 1182.9 | 1222.8 | 5 and 6 | 70.433 | 2 | <.001 |
| h. PC*group, type of task, and pre-task affect model (**model 7**)^a^ | 620 | -573.68 | 1167.4 | 1211.7 | 6 and 7 | 11.717 | 1 | .001 |
| h. PC*group, type of task, pre-task affect, and covariates model (model 8) | 620 | -573.44 | 1172.9 | 1230.5 | 7 and 8 | 0.473 | 3 | .925 |
| *Note.* AS = parental autonomy support; PC = parental psychological control; L/U = parental listening and understanding; C/D = parental criticism and dominance; HC = families with a healthy control adolescent; DEP = families with an adolescent with current MDD/dysthymia; PSI = problem solving interaction task; EPI = event planning interaction task; REM = reminiscence interaction task. Covariates: parental sex, adolescent sex and age. Sex: 0 = female, 1 = male; Group: 0 = HC, 1 = DEP. Type of task model: dummy coding with PSI as reference category.  ^a^ Model fit comparison could not run on model 6 and 7 with adolescent affect as the dependent variable, because of missing data for pre-task affect (*n* = 3 observations). In order to compute model fit statistics in comparing model 6 and 7 with adolescent affect as the dependent variable, cases with missing data for pre-task affect were excluded in the comparison of these models. | | | | | | | | |

| **Supplementary Table 9**  *Results Multilevel Analyses on the Effect of Group on Observed AS and PC (H2.1) and Perceived L/U and C/D (H2.2)* | | | | | | | | | | |
| --- | --- | --- | --- | --- | --- | --- | --- | --- | --- | --- |
|  |  |  | Observed AS | | Observed PC | | Perceived L/U | | Perceived C/D | |
|  |  |  | Estimate (*SE*) | *p* | Estimate (*SE*) | *p* | Estimate (*SE*) | *p* | Estimate (*SE*) | *p* |
| Fixed effects | | |  |  |  |  |  |  |  |  |
|  | Intercept | | -0.175 (.080) | .029 | 0.275 (.074) | <.001 | -0.200 (.085) | .021 | 1.387 (.749) | .066 |
|  | Group (HC v. DEP) | | -0.184 (.117) | .120 | 0.083 (.100) | .410 | -0.431 (.145) | .004 | 0.136 (.163) | .406 |
|  | Type of task | |  |  |  |  |  |  |  |  |
|  |  | EPI v. PSI | 0.221 (.082) | .007 | -0.467 (.086) | <.001 | 0.529 (.056) | <.001 | -0.782 (.072) | <.001 |
|  |  | REM v. PSI | 0.487 (.082) | <.001 | -0.433 (.086) | <.001 | 0.486 (.056) | <.001 | -0.829 (.073) | <.001 |
|  |  | REM v. EPI^a^ | 0.226 (.082) | .001 | 0.034 (.086) | .691 | -0.043 (.056) | .446 | -0.047 (.073) | .517 |
|  | Group*Type of task | |  |  |  |  |  |  |  |  |
|  |  | Group*EPI (v. PSI) |  |  |  |  |  |  | 0.057 (.133) | .669 |
|  |  | Group*REM (v. PSI) |  |  |  |  |  |  | 0.293 (.133) | .028 |
|  |  | Group*EPI (v. REM) ^a^ |  |  |  |  |  |  | 0.236 (.133) | .076 |
|  | Biological sex parent | |  |  |  |  |  |  | -0.160 (.070) | .023 |
|  | Biological sex adolescent | |  |  |  |  |  |  | -0.232 (.142) | .106 |
|  | Age adolescent | |  |  |  |  |  |  | -0.043 (.047) | .363 |
| Random effects | | |  |  |  |  |  |  |  |  |
|  | Between person variance | | .114 |  | .178 |  | .302 |  | .110 |  |
|  | Within person variance | | .704 |  | .772 |  | .323 |  | .380 |  |
|  | Between family variance | | .136 |  | .002 |  | .274 |  | .347 |  |
| Intraclass correlation | | |  |  |  |  |  |  |  |  |
|  | Person level | | .10 |  | .16 |  | .28 |  | .06 |  |
|  | Family level | | .14 |  | .00 |  | .31 |  | .36 |  |
| *Note.* *N* = 148 parents; *n* = 442 observations of observed parenting; *n* = 437 observations of perceived parenting. AS = parental autonomy support; PC = parental psychological control; L/U = parental listening and understanding; C/D = parental criticism and dominance; HC = families with a healthy control adolescent; DEP = families with an adolescent with current MDD/dysthymia; PSI = problem solving interaction task; EPI = event planning interaction task; REM = reminiscence interaction task. Sex: 0 = female, 1 = male. Group: 0 = HC, 1 = DEP.  ^a^ Task difference REM v. EPI are tested in a separate model. Output for this variable is based on the model including EPI as the reference category in dummy coding. Whereas all other output is based on the model including PSI as the reference category in dummy coding. | | | | | | | | | | |

| **Supplementary Table 10**  *Results Multilevel Analyses on the Relation between Observed AS and PC, and Perceived L/U and C/D (H2.3)* | | | | | | | | | | |
| --- | --- | --- | --- | --- | --- | --- | --- | --- | --- | --- |
|  |  |  | Observed AS | | | | Observed PC | | | |
|  |  |  | Perceived L/U | | Perceived criticism | | Perceived L/U | | Perceived C/D | |
|  |  |  | Estimate (*SE*) | *p* | Estimate (*SE*) | *p* | Estimate (*SE*) | *p* | Estimate (*SE*) | *p* |
| Fixed effects | | |  |  |  |  |  |  |  |  |
|  | Intercept | | -0.185 (.084) | .030 | 1.414 (.749) | .061 | -0.174 (.084) | .041 | 1.362 (.745) | .070 |
|  | Observed parenting | | 0.069 (.037) | .066 | -0.131 (.039) | .001 | -0.082 (.036) | .023 | 0.113 (.038) | .003 |
|  | Group (HC v. DEP) | | -0.412 (.143) | .005 | 0.237 (.144) | .104 | -0.420 (.142) | .004 | 0.241 (.144) | .095 |
|  | Observed parenting*Group | | 0.062 (.063) | .322 | 0.079 (.065) | .223 | -0.067 (.060) | .264 | 0.011 (.062) | .865 |
|  | Type of task | |  |  |  |  |  |  |  |  |
|  |  | EPI v. PSI | 0.512 (.056) | <.001 | -0.741 (.060) | <.001 | 0.486 (.057) | <.001 | -0.714 (.062) | <.001 |
|  |  | REM v. PSI | 0.446 (.058) | <.001 | -0.689 (.062) | <.001 | 0.446 (.057) | <.001 | -0.693 (.062) | <.001 |
|  |  | REM v. EPI^a^ | -0.066 (.056) | .243 | 0.053 (.061) | .390 | -0.040 (.056) | .473 | 0.021 (.060) | .729 |
|  | Biological sex parent | |  |  | -0.148 (.069) | .035 |  |  | -0.158 (.069) | .023 |
|  | Biological sex adolescent | |  |  | -0.237 (.142) | .099 |  |  | -0.234 (.142) | .099 |
|  | Age adolescent | |  |  | -0.048 (.047) | .309 |  |  | -0.046 (.047) | .335 |
| Random effects | | |  |  |  |  |  |  |  |  |
|  | Between person variance | | .291 |  | .110 |  | .286 |  | .104 |  |
|  | Within person variance | | .320 |  | .375 |  | .318 |  | .376 |  |
|  | Between family variance | | .264 |  | .348 |  | .266 |  | .347 |  |
| Intraclass correlation | | |  |  |  |  |  |  |  |  |
|  | Person level | | .28 |  | .06 |  | .28 |  | .06 |  |
|  | Family level | | .31 |  | .36 |  | .31 |  | .36 |  |
| *Note.* *N* = 148 parents; *n* = 442 observations of observed parenting; *n* = 437 observations of perceived parenting. AS = parental autonomy support; PC = parental psychological control; L/U = parental listening and understanding; C/D = parental criticism and dominance; HC = families with a healthy control adolescent; DEP = families with an adolescent with current MDD/dysthymia; PSI = problem solving interaction task; EPI = event planning interaction task; REM = reminiscence interaction task. Sex: 0 = female, 1 = male. Group: 0 = HC, 1 = DEP.  ^a^ Task difference REM v. EPI are tested in a separate model. Output for this variable is based on the model including EPI as the reference category in dummy coding. Whereas all other output is based on the model including PSI as the reference category in dummy coding. | | | | | | | | | | |

| **Supplementary Table 11**  *Results Multilevel Analyses on the Relation between Observed AS and PC, and Adolescent Positive and Negative Affect (H2.3)* | | | | | | | | | | |
| --- | --- | --- | --- | --- | --- | --- | --- | --- | --- | --- |
|  |  |  | Observed AS | | | | Observed PC | | | |
|  |  |  | Positive affect | | Negative affect | | Positive affect | | Negative affect | |
|  |  |  | Estimate (*SE*) | *p* | Estimate (*SE*) | *p* | Estimate (*SE*) | *p* | Estimate (*SE*) | *p* |
| Fixed effects | | |  |  |  |  |  |  |  |  |
|  | Intercept | | 0.203 (.068) | .004 | -0.226 (.064) | .001 | 0.215 (.078) | .007 | -0.221 (.063) | .001 |
|  | Observed parenting | | -0.021 (.032) | .526 | -0.012 (.032) | .719 | 0.017 (.030) | .567 | -0.015 (.031) | .631 |
|  | Group (HC v. DEP) | | -0.930 (.121) | <.001 | 0.833 (.110) | <.001 | -1.087 (.139) | <.001 | 0.810 (.107) | <.001 |
|  | Observed parenting*Group | | 0.029 (.054) | .589 | -0.037 (.052) | .486 | -0.067 (.050) | .177 | 0.122 (.050) | .015 |
|  | Type of task | |  |  |  |  |  |  |  |  |
|  |  | EPI v. PSI | 0.426 (.068) | <.001 | -0.297 (.052) | <.001 | 0.449 (.049) | <.001 | -0.296 (.054) | <.001 |
|  |  | REM v. PSI | -0.182 (.057) | .002 | 0.190 (.055) | .001 | -0.092 (.050) | .069 | 0.191 (.055) | .001 |
|  |  | REM v. EPI^a^ | -0.607 (.055) | <.001 | 0.487 (.054) | <.001 | -0.541 (.050) | <.001 | 0.486 (.054) | <.001 |
|  | Pre-task affect | | 0.178 (.040) | <.001 | 0.270 (.037) | <.001 | -0.039 (.040) | .324 | 0.290 (.037) | <.001 |
| Random effects | | |  |  |  |  |  |  |  |  |
|  | Between person variance | | .044 |  | .009 |  | .077 |  | <.001 |  |
|  | Within person variance | | .266 |  | .280 |  | .237 |  | .285 |  |
|  | Between family variance | | .229 |  | .187 |  | .329 |  | .177 |  |
| Intraclass correlation | | |  |  |  |  |  |  |  |  |
|  | Person level | | .06 |  | .06 |  | .06 |  | .06 |  |
|  | Family level | | .63 |  | .66 |  | .63 |  | .66 |  |
| *Note.* *N* = 148 parents; *n* = 442 observations of observed parenting; *n* = 437 observations of adolescent affect. AS = parental autonomy support; PC = parental psychological control; L/U = parental listening and understanding; C/D = parental criticism and dominance; HC = families with a healthy control adolescent; DEP = families with an adolescent with current MDD/dysthymia; PSI = problem solving interaction task; EPI = event planning interaction task; REM = reminiscence interaction task. Sex: 0 = female, 1 = male. Group: 0 = HC, 1 = DEP.  ^a^ Task difference REM v. EPI are tested in a separate model. Output for this variable is based on the model including EPI as the reference category in dummy coding. Whereas all other output is based on the model including PSI as the reference category in dummy coding. | | | | | | | | | | |

**Supplementary References**

Allen, J. P., Hauser, S. T., Eickholt, C., Bell, K. L., & O’Connor, T. G. (1994). Autonomy and relatedness in family interactions as predictors of expressions of negative adolescent affect. *Journal of Research on Adolescence, 4*(4), 535–552. <https://doi.org/10.1207/s15327795jra0404_6>

Barber, B. K. (1996). Parental psychological control: Revisiting a neglected construct. *Child Development, 67*(6), 3296–3319. <https://doi.org/10.1111/j.1467-8624.1996.tb01915.x>

Barber, B. K., Stolz, H. E., Olsen, J. A., Collins, W. A., & Burchinal, M. (2005). Parental support, psychological control, and behavioral control: Assessing relevance across time, culture, and method. *Monographs of the Society for Research in Child Development, 70*(4), 1–147.

Deci, E. L., & Ryan, R. M. (2000). The “what” and “why” of goal pursuits: Human needs and the self-determination of behavior. *Psychological Inquiry, 11*(4), 227–268. <https://doi.org/10.1207/S15327965PLI1104_01>

Donatelli, J.-A. L., Bybee, J. A., & Buka, S. L. (2007). What do mothers make adolescents feel guilty about? Incidents, reactions, and relation to depression. *Journal of Child and Family Studies, 16*(6), 859–875. <https://doi.org/10.1007/s10826-006-9130-1>

Elzy, M. B. (2013). *Emotional invalidation: An investigation into its definition, measurement, and effects* [Doctoral Dissertation, University of South Florida]. USF Tampa Graduate Theses and Dissertations. <https://digitalcommons.usf.edu/etd/4670>

Grolnick, W. S., Ryan, R. M., & Deci, E. L. (1991). Inner resources for school achievement: Motivational mediators of children’s perceptions of their parents. *Journal of Educational Psychology, 83*(4), 508–517. <https://doi.org/10.1037/0022-0663.83.4.508>

Hauser Kunz, J., & Grych, J. H. (2013). Parental psychological control and autonomy granting: Distinctions and associations with child and family functioning. *Parenting, 13*(2), 77–94. <https://doi.org/10.1080/15295192.2012.709147>

Kaufman, J., Birmaher, B., Brent, D., Rao, U., & Ryan, N. (1996). *Kiddie-sads-present and lifetime version (K-SADS-PL).* Pittsburgh, PA: University of Pittsburgh, School of Medicine.

Kroenke, K., Spitzer, R. L., & Williams, J. B. W. (2001). The PHQ-9. *Journal of General Internal Medicine, 16*(9), 606–613. <https://doi.org/10.1046/j.1525-1497.2001.016009606.x>

Kullberg, M.-L., Maciejewski, D., van Schie, C. C., Penninx, B. W. J. H., & Elzinga, B. M. (2020). Parental bonding: Psychometric properties and association with lifetime depression and anxiety disorders. *Psychological Assessment, 32*(8), 780–795. <https://doi.org/10.1037/pas0000864.supp>

Lansford, J. E., Laird, R. D., Pettit, G. S., Bates, J. E., & Dodge, K. A. (2014). Mothers’ and fathers’ autonomy-relevant parenting: Longitudinal links with adolescents’ externalizing and internalizing behavior. *Journal of Youth and Adolescence, 43*(11), 1877–1889. <https://doi.org/10.1007/s10964-013-0079-2>

Little, R. J. A. (1988). A test of missing completely at random for multivariate data with missing values. *Journal of the American Statistical Association, 83*(404), 1198–1202. <https://doi.org/10.1080/01621459.1988.10478722>

Mageau, G. A., Bureau, J. S., Ranger, F., Allen, M.-P., & Soenens, B. (2016). The role of parental achievement goals in predicting autonomy-supportive and controlling parenting. *Journal of Child and Family Studies, 25*(5), 1702–1711. <https://doi.org/10.1007/s10826-015-0341-1>

Reichart, C. G., Wals, M., & Hillegers, M. (2000). *Vertaling K-SADS.* Utrecht: HC Rümke Groep.

Rogers, K. N., Buchanan, C. M., & Winchell, M. E. (2003). Psychological control during early adolescence: Links to adjustment in differing parent/adolescent dyads. *The Journal of Early Adolescence, 23*(4), 349–468. <https://doi.org/10.1177/0272431603258344>

Ryan, R. M., & Deci, E. L. (2000). Self-determination theory and the facilitation of intrinsic motivation, social development, and well-being. *American Psychologist, 55*(1), 68–78. <https://doi.org/10.1037/0003-066X.55.1.68>

Sheehan, D. V., Lecrubier, Y., Sheehan, K. H., Amorim, P., Janavs, J., Weiller, E., & Dunbar, G. C. (1998). The Mini-International Neuropsychiatric Interview (M.I.N.I.): The development and validation of a structured diagnostic psychiatric interview for DSM-IV and ICD-10. *Journal of Clinical Psychiatry, 59*, 22–33.

Soenens, B., & Vansteenkiste, M. (2010). A theoretical upgrade of the concept of parental psychological control: Proposing new insights on the basis of self-determination theory. *Developmental Review, 30*(1), 74–99. <https://doi.org/10.1016/j.dr.2009.11.001>

Soenens, B., Vansteenkiste, M., Lens, W., Luyckx, K., Goossens, L., Beyers, W., & Ryan, R. M. (2007). Conceptualizing parental autonomy support: Adolescent perceptions of promotion of independence versus promotion of volitional functioning. *Developmental Psychology, 43*(3), 633–646. <https://doi.org/10.1037/0012-1649.43.3.633>

Tam, C. L., & Yeoh, S. H. (2008). Parental bonding and parent-child relationship among tertiary students. *Sunway Academic Journal, 5*, 111–127.

Wuyts, D., Soenens, B., Vansteenkiste, M., & Van Petegem, S. (2018). The role of observed autonomy support, reciprocity, and need satisfaction in adolescent disclosure about friends. *Journal of Adolescence, 65*, 141–154. <https://doi.org/10.1016/j.adolescence.2018.03.012>
